# A review of the modern principles and applications of solid-phase extraction techniques in chromatographic analysis

**DOI:** 10.1007/s44211-022-00190-8

**Published:** 2022-10-05

**Authors:** Mohamed E. I. Badawy, Mahmoud A. M. El-Nouby, Paul K. Kimani, Lee W. Lim, Entsar I. Rabea

**Affiliations:** 1 Department of Pesticide Chemistry and Technology, Laboratory of Pesticide Residues Analysis, Faculty of Agriculture, Alexandria University, Aflatoun St., 21545-El-Shatby, Alexandria, Egypt; 2 Department of Engineering, Graduate School of Engineering, Gifu University, 1-1 Yanagido, Gifu, 501-1193 Japan; 3 International Joint Department of Materials Science and Engineering Between National University of Malaysia and Gifu University, Graduate School of Engineering, Gifu University, 1-1 Yanagido, Gifu, 501-1193 Japan; 4 Department of Plant Protection, Faculty of Agriculture, Damanhour University, Damanhour, 22516 Egypt

**Keywords:** SPE, Separation technique, Cleanup, Chromatographic analysis

## Abstract

Analytical processes involving sample preparation, separation, and quantifying analytes in complex mixtures are indispensable in modern-day analysis. Each step is crucial to enriching correct and informative results. Therefore, sample preparation is the critical factor that determines both the accuracy and the time consumption of a sample analysis process. Recently, several promising sample preparation approaches have been made available with environmentally friendly technologies with high performance. As a result of its many advantages, solid-phase extraction (SPE) is practiced in many different fields in addition to the traditional methods. The SPE is an alternative method to liquid–liquid extraction (LLE), which eliminates several disadvantages, including many organic solvents, a lengthy operation time and numerous steps, potential sources of error, and high costs. SPE advanced sorbent technology reorients with various functions depending on the structure of extraction sorbents, including reversed-phase, normal-phase, cation exchange, anion exchange, and mixed-mode. In addition, the commercial SPE systems are disposable. Still, with the continual developments, the restricted access materials (RAM) and molecular imprinted polymers (MIP) are fabricated to be active reusable extraction cartridges. This review will discuss all the theoretical and practical principles of the SPE techniques, focusing on packing materials, different forms, and performing factors in recent and future advances. The information about novel methodological and instrumental solutions in relation to different variants of SPE techniques, solid-phase microextraction (SPME), in-tube solid-phase microextraction (IT-SPME), and magnetic solid-phase extraction (MSPE) is presented. The integration of SPE with analytical chromatographic techniques such as LC and GC is also indicated. Furthermore, the applications of these techniques are discussed in detail along with their advantages in analyzing pharmaceuticals, biological samples, natural compounds, pesticides, and environmental pollutants, as well as foods and beverages.

**Graphical abstract:**

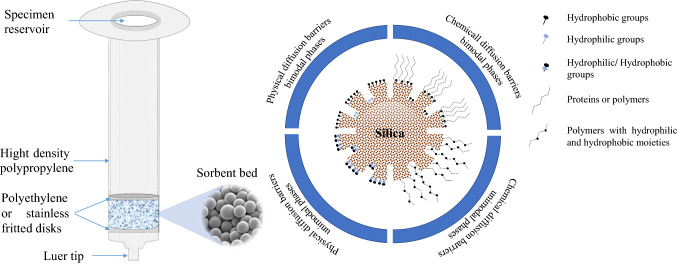

## Theory and principles of SPE

**Fig. 1:**
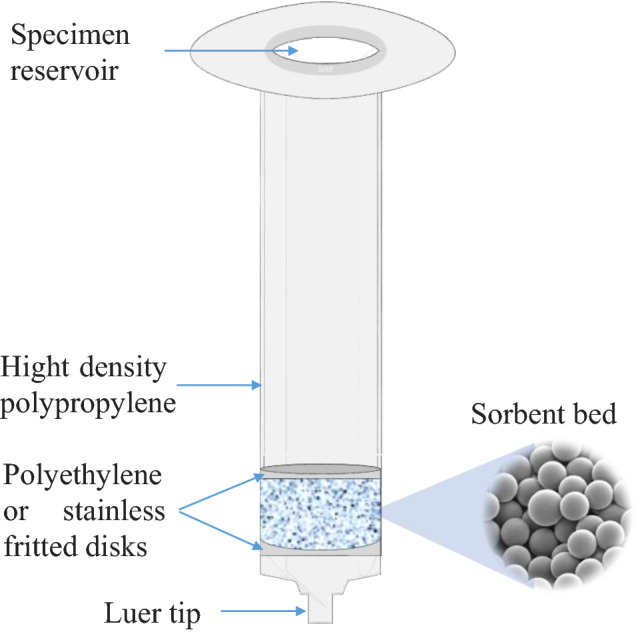
Schematic diagram of SPE cartridge

Solid-phase extraction (SPE) is an effective way to prepare samples in chemistry. A sample collection and analysis gap can be closed using SPE as one of the methods. It is rarely used with no further preparation steps, such as dilution or pH adjustment [1–3]. Similar to liquid–liquid extraction (LLE), SPE is based on similar principles. The distribution of analytes or solutes between two phases is similar in both procedures. SPE does not require mixing of two liquid phases, as in LLE, but rather involves dispersion of the analyte between two liquid phases (sample medium and adsorbent). The liquid sample is passed through adsorbent particles to which the analytes have a greater affinity than the bulk liquid. Subsequently, the analytes are extracted by elution with an appropriate solvent (Fig. 1). This extraction method simplifies the analysis by removing much of the sample matrix. Due to its ease and economy in terms of time and solvents, SPE is becoming more prevalent for preconcentration of analyte and matrix removal than LLE. In addition, LLE is inefficient at extracting polar compounds, time-consuming and labor-intensive, tends to form emulsions, requires a large amount of solvents to evaporate, and uses chemical disposal of potentially toxic or explosive substances [4, 5]. This sample processing technique has become the method of choice in many environmental analytical applications and has been gradually included in standardized procedures in the past decade. Therefore, the SPE technique became familiar to a broad analytical public. However, there are a few drawbacks of SPE technologies. These drawbacks have been overcome by discovering new microextraction techniques such as magnetic SPE (MSPE), solid-phase microextraction (SPME), and kinetic adsorption extraction [4].

## A brief history of SPE

Due to its many advantages over other traditional methods, SPE was applied for the first time during the 1940s [6], and has quickly grown in many applications during the 1970s. Animal charcoal was probably the first adsorbent in SPE column for removing pigments from reaction mixtures. During the 1970s, SPE began to be recognized as a scientific technique. Since its inception in 1968, three phases of sample preparation have been developed (1968–1977, 1977–1989, and 1989-present). During these phases, sorbent products have grown significantly in popularity. At the same time, technological advances have changed the types and forms of sorbent products. Several synthetic polymers (e.g., styrenedivinylbenzene resins) were used in the first publications of SPE applications [7].

The first experimental applications of SPE beginning in the 1950s were the analysis of organic traces in water samples. Hundreds of articles have been published over the past few years in scientific journals describing SPE as a water analytical tool and method for quantifying organic compounds [3, 8]. The introduction of pre-filled cartridges/columns containing silica sorbents in October 1977 made the procedure more convenient and began another phase of development. In May 1978, this technology appeared on the cover of laboratory equipment. In addition, the first article using SPE on silica was published in the bonding process [9], which described the Sep Pak™ C_18_ to clean histamine from wine. The introduction of stable and covalently bound chromatographic adsorbents, especially those with a reverse phase, has opened applications in the environmental, clinical and pharmaceutical markets.

Because of its carbonaceous nature, C18 can be used for HPLC or SPE processes that do not require polar interactions. SPE sorbents are commonly packed into cartridges or columns, and are typically of the bead shape [10–12]. Also of current development and use is incorporating sorbent particles into disk formats [13, 14]. Due to small porous particles and rapid mass transport, the disk format has been able to produce good extraction recoveries with high flow rates.

In 1989, SPE disks or membranes were introduced, and another stage in SPE development began [15, 16]. In these disks, an absorbent material is placed between Teflon or fiberglass pads, or within the matrix. Using this design, an extremely short and extremely accurate SPE cartridge is created. The use of rigid polymeric monoliths as a stationary phase alternative for liquid chromatography became common in the 1990s [17, 18]. Shortly after, monolithic polymer stationary phases made their way into the field of SPE. The first monolithic polymer incorporated into an SPE device was poly(styrene-co-divinylbenzene) (PS-DVB) [19]. Huck et al. investigated the recovery of thirteen pesticides, using both PS-BVD and octadecyl silica (ODS) phases [20]. PS-DVB was found to achieve an average recovery of 77% for all compounds, compared to 69% for ODS. PS-DVD copolymers have proven to be very effective in recovering non-polar compounds due to their inherent hydrophobicity. Monolithic sorbents were also excellent materials for high-throughput SPE experiments. Monolithic SPE sorbent can deliver superior mass transfer characteristics due to the highly interconnected pores and excellent permeability. However, polar analytes were, on average, less well retained by PS-DVB copolymers than non-polar analytes [21]. Therefore, multi-functional polymer-based sorbents have been introduced to enhance the retention of the polar analyte through hydrophilic interactions [20].

## SPE configurations

SPE is a versatile technology for the purification, separation, and concentration of analytes from a sample solution matrix using a sorbent bed by flow-through equilibrium. A wide range of SPE extraction configurations, including SPE cartridges, disks, multi-well SPE, SPME, and in-tube SPME (IT-SPME), are designed to be process-analytical compatible with ease of use and cost in mind. However, the cost can be a significant issue with many samples [3, 14]. Therefore, the comparison between different SPE techniques is presented in Table 1.

**Table 1:** Comparison of some aspects of SPE techniques

Parameter	Cartridge	PT-SPE	Disk	Multi-well SPE	SPME	IT-SPME
Classification	Exhaustive flow-through equilibrium and pre-equilibrium				Non-exhaustive batch equilibrium and pre-equilibrium	Non-exhaustive flow-through equilibrium and pre-equilibrium
Weight of sorbent	4–30 mg	4–400 µg	4– 200 mg	3–200 mg	–	–
Applicable volume	500–50 mL	0.5–1 mL	0.5–1 L	0.65–2 mL	–	–
Application	Wide variety of sample matrices	Biological samples	Substantial samples	Biological samples	Environmental and biomedical samples	Environmental and biomedical samples
Benefits	Easy to assemble in the laboratory Wide range of uses Low cost Possibility of storage of analytes enriched on solid sorbent	Simplicity and shorter extraction time High sensitivity and recovery factors A small quantity of elution volume Conditioning steps are not required Amenable to automation by available tools (micropipette)	Operated with a smaller elution volume Greater cross-sectional area Fast flow rates Smaller void volume Ignored the filtration of the extract Slighter extraction period for substantial samples	Rapid preparation of a large number of samples Less labor and time-consuming Less solvent waste Fast flow rates Amenable to automation	Green extraction Rapid extraction Miniaturized technique Low analysis cost Easiness of automation Friendly-eco	Miniaturized technique Large volume samples Compatible with analytical instruments
Limitations	Partially small cross-section Sluggish flow rate Tremendous unavailable rented volume Plugging Channeling Costly with a large number of samples	Restricted flow rates and plugging A large amount of plastic waste	Decrease in breakthrough volume Small samples would be lost Costly	Due to open-bed configuration, the technique is unsuitable for volatile analytes	Low adsorption capacity Limited effectiveness	A large amount of solvent needed

### SPE cartridge

The cartridges are the most common disposable format. It comprises a high-density polypropylene syringe filed by different amounts of the sorbent bed between two frits (Fig. 1). The sorbent amount differed from milligrams to several grams depending on the applicable sample volume (500 µL up to 50 mL). The sample flows through the sorbent bed by pressure from the top using a piston (positive pressure) or a vacuum (pressure reduction). SPE can retain approximately 5% of its sorbent mass without significant breakthrough. Thus, the most popular configurations are the 500 mg SPE cartridges of packing in 3 and 5 mL syringe barrels, which are required to purify the large volume of environmental samples (expected to retain ~ 25 mg of the analytes). However, the smaller mass (100 mg of sorbent in a 1 mL syringe) cartridges are necessary for fast cleanup, improving the analysis sensitivity by reducing elution volume, especially for biological samples. In addition, fully robotic (automated) systems are introduced for the batches of samples using vacuum manifolds [22, 23].

### Pipette-tips SPE (PT-SPE)

**Fig. 2:**
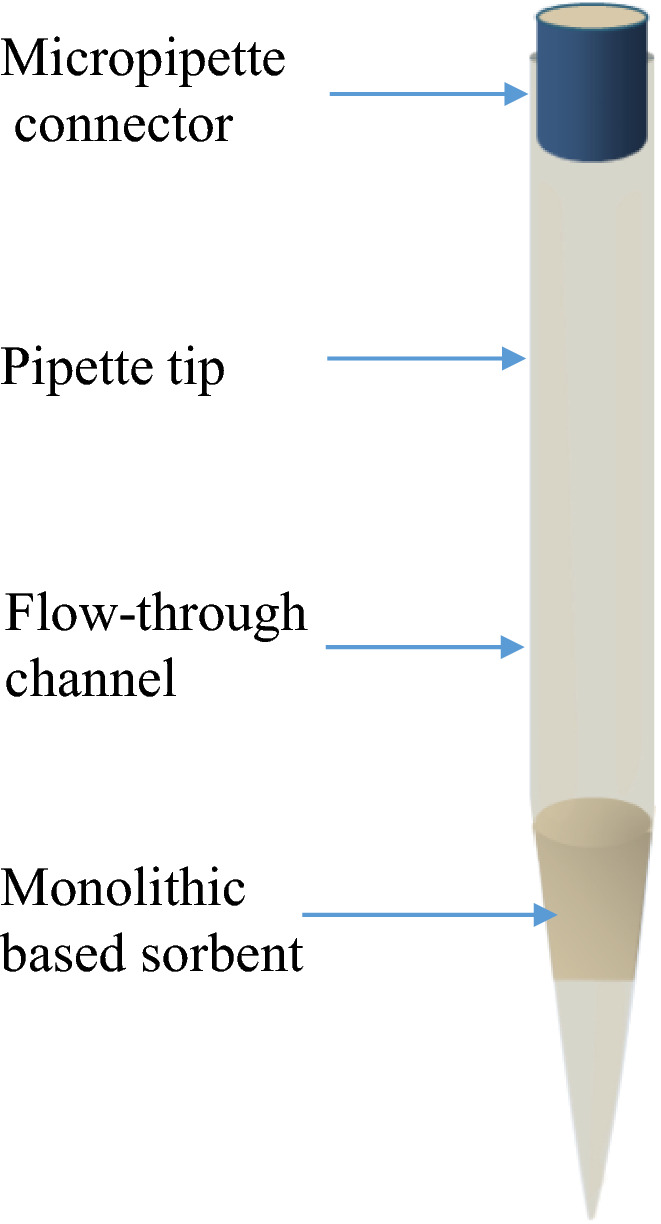
Schematic diagram of PT-SPE

PT-SPE is a new miniaturized format of SPE to facilitate automated systems using available tools in many laboratories. The use of this technique for purifying and concentrating proteins and peptides has become essential to the study of genomics, proteomics, and metabolism [24]. Many different shapes of PT-SPE are produced to adapt quickly to the liquid-handling systems, encompassing pipette tips. Ansys Technologies (Lake Forest, California, USA) introduced the sorbent-immobilized supported PT-SPE format in 1998 [25]. Therefore, it was an obvious extension of the technologies to investigate using sorbent-filled pipette tips for SPE. A pipette tip carrying the PT-SPE material has a fine slit at its bottom (1–2 µm in width) that allows the liquid phase to pass through while the chromatographic material (20–30 µm) remains in the tip. It also reduces dead volume since no filter is needed. In addition, sampling is faster and cuts down on samples lost [26]. Recently, the PT-SPE was fabricated based on a porous monolithic sorbent bed without a supporter bed (Fig. 2). PT-SPE immediately gained recognition among analytical chemists because reducing the sorbent amount contributed significantly to reducing the used amount of organic solvents, which lowered the costs and performed Eco-Friendly extraction and purification quickly [26, 27].

### Disks

The disks are similar to the SPE cartridge, while the disk is disposable and placed in a reusable holder. However, disks were attempted with a much greater cross-sectional area ranging from 4 to 96 mm); furthermore, much smaller sorbent particles (8 μm) were packed in the cartridges (40 μm). These differences help increase the surface area for effective and rapid extraction of analytes, especially in large volumes of low-concentration environmental samples. In addition, the disks may be used to remove suspended particulate and impurities while letting the analytes pass through the disk [28, 29]. The most commonly used disk size is 47 mm, adequate for water samples of volumes from 0.5 to 1 L. The disk is made commercially in two generic versions, based on the combined sorbent method, including immobilizing the sorbent on a polymer or fiberglass and the sorbent material packed between two glass filters. Polymer immobilized sorbent is frequently used for environmental samples. This type consists of polytetrafluoroethylene (PTEF) microfibers (10%) that are loaded by 8 µm particles in the ratio of approximately 90% (w/w) in diameter of 25–90 mm [28]. The fiberglass-based disk contains the bonded silica particles (10-30 µm) woven into the fiberglass, which provides a more porous filter than the Empore disk. The fixed sorbent bed in diameter of 10 µm packed between two laminar fiberglasses (Speedisk) was introduced in 1998 by J. T Beker. In a vacuum manifold or filtration flask, the Speedisk is easy to use and contributes to higher recovery of analytes by achieving a higher flow value of the sample [25].

### Multi-well SPE plates

The microtiter plate is the common name for the Multi-well SPE plates. Deferent Kinds of Multi-well SPE plates, including 96-Well SPE Plates (8 × 12 well), 384-Well SPE Plates (16 × 24 well), and 1536-well plates (32 × 48 well), are widely used for processing large numbers of samples in automated instruments using the vacuum manifold. The automated 96-workstation SPE was introduced in 1996 to significantly improve the automation of a large number of samples [30]. Since then, various robotic 96, 384, and 1536-well-formats have been commercialized. Each well has a small SPE cartridge (0.65–2 mL) consisting of packing sorbent (3–200 mg) between the top and bottom frits. Multi-well plates can be classified into two types: fixed and flexible formats. The Fixed plate has immutable cartridge volume and a fixed quantity of the stationary phase. In contrast, the elastic well plates have small removable SPE cartridges that fit tightly into the plastic plate frame [3]. The success of Multi-well format SPE has also led to the best robotic control of the sample and solvent manipulation, which increases the precision and accuracy compared with manual methods.

### Solid-phase microextraction (SPME)

**Fig. 3:**
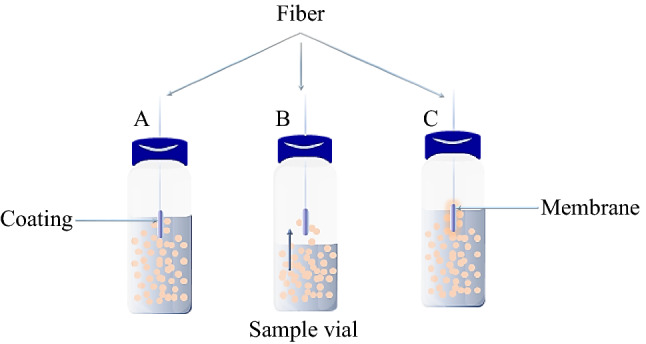
Different operation modes of SPME. **A** Direct extraction; **B** headspace SPME; and **C** membrane-protected SPME

SPME is an environmentally friendly (solvent-free) sample preparation process introduced in 1990 [31]. The SPME approach is sometimes confused with SPE. In this technique, the analytes are equilibrated with both the matrix and the fiber, allowing for non-exhaustive microextraction. The microextraction mechanism consists of exposing a small amount of extracting phase (fused-silica fiber) coated with a thin layer (7–100 μm thick) of immobilized polymer or a solid adsorbent [30, 32]. SPME involves three basic operation modes: direct extraction, headspace SPME, and membrane-protected SPME (Fig. 3). In the direct extraction mode, and headspace, an adsorbent polymer coats the fiber rod. However, the direct extraction, the fiber is inserted into the sample solution, and the analytes are extracted directly from the sample solution to the coating sorbent. While the headspace mode, the fiber is placed above the sample solution to remove the volatile compounds, which is essential to reducing macromolecules’ interference effect. The membrane-protected SPME is the most trendy used due to the positive effect of the selective-access membrane to protect the coated polymer from interferences. It is possible to perform SPME techniques manually or automatically, coupled with chromatographic systems efficiently.

### In-tube solid-phase microextraction (IT-SPME)

IT-SPME, also known as capillary microextraction, is one of the non-exhaustive flow-through equilibrium and pre-equilibrium techniques that have been developed since its introduction in 1997 by Eisert and Pawliszyn [33]. IT-SPME uses capillary tubes as extraction devices and is classified based on the packed materials involving coated tube IT-SPME, sorbent packing, fiber packing, and monolith. By coupling IT-SPME on-line with the LC system in real-time, it is capable of fully automating the entire process from sample preparation to separation, and detection of targets analytes. The vast range of applications of IT-SPME technology is expanding in various areas, and the expected future development is to provide safe and eco-friendly green sample preparation methods [34, 35].

## SPE packing materials based on the retention mode

It is necessary to know the different SPE strategies, depending on the target of the extraction. Three common SPE strategies are bind and elute, interference removal, and fractionation [36]. The bind and elute strategy is the most common strategy consisting of two consequence processes; bind the analytes with the stationary phase, and unwanted matrices are washed out, then change the solvent system to elute the analytes from the sorbent. The general technical steps of the SPE procedure are started by pretreatment of the sample depending on the properties of the analyte, the sample matrix, and the nature of the retention mechanism, such as pH adjustment, centrifugation, filtration, and dilution. They were then conditioning the SPE with a suitable moisture solvent. Next, they correlated functional groups to activate the coherent interaction. Next, the equilibration step should occur by treating the sorbent with a similar solution (in polarity, pH, etc.) to maximize retention before loading the sample matrix. These steps are the same in all strategies. They finally eluted the analytes of interest with an appropriate solvent to overcome the retention interactions between sorbent and analytes of interest. The interference removal strategy is like a chemical filtration process. The unwanted matrix components bind strongly on the stationary phase, allowing analytes to pass through the sample loading stage. The fractionation strategy seems like the bind elute strategy when extracting different compounds by retaining all the analytes and sequentially eluting other analytes by modifying eluant pH or the percentage of organic solvent.

In general, the SPE is performed using either silica-based or polymer-based sorbents. The SPE sorbent phase is mostly similar to that commonly packed into specific HPLC columns. Thus, depending on the fundamental principles of standard chromatographic processes, the nature of the SPE sorbent phase can vary in the bonded functional group based on retention mechanisms, the analyte, the sample conditions, and the solvent used in conditioning and elution steps (Table 2). In addition, the most frequently exploited mechanisms in SPE are classified into three main mechanisms, including normal-phase, reversed-phase, and ion-exchange. Recently, the multimode mechanisms were obtained by combining more than one mode in the same sorbent materials and the promising sorbent, including molecularly imprinted polymers (MIPs) and restricted access media (RAM).

**Table 2:** Various retention modes and typical steps that carried out during the SPE process

Sorbent phase type		Bed sorbent	Structure of bonded phases	Bonded function	Target analyte	Solvents		
						Conditioning	Rinse	Elution
Normal-phase		Amino (-NH_2_)	-(CH_2_)NH_2_	Polar interactions Hydrogen bonding Dipolar attraction	Exhibits polar functionalities (hydroxyl, carbonyls, amines, double bonds, compounds with heteroatoms)	Nonpolar solvent (hexane, chloroform)	Nonpolar solvent	Strong polar eluent (such as methanol)
		Diol (C–OH C–OH)	Spaced-bonded propanediol					
		Cyano (-CN)	-(CH_2_)_3_CN	Polar interactions Adsorption				
		Silica gel	-SiOH					
		Florisil	Mg_2_SiO_3_					
		Alumina	Al_2_O_3_					
Reversed-phase		Triacontyl	-C_30_H_61_	Adsorption/hydrophobic interaction van der Waals forces	Slightly non-polar Moderately non-polar Nonpolar	MeOH/H_2_O ACN/H_2_O with appropriate pH	Polar solvent With appropriate pH	Strong non-polar (hexane, chloroform) Polar to semi-polar (methanol)
		Octadecyl (C_18_)	-(CH_2_)_17_CH_3_					
		Octyl (C_8_)	-(CH_2_)_7_CH_3_					
		Ethyl (C_2_)	-CH_2_CH_3_					
		Cyclohexyl	CH_2_-Cyclohexyl					
		Phenyl	CH_2_-Phenyl					
Ion exchange	SCX	Sulfonic	Propanesulfonic acid	Electrostatic attraction (cation exchange-ionic base)	Positive charge analytes	Water or buffer pH > 7 (pH = pKa − 2)	pH = pKa-2	Eluent with ionic strength (pH = pKa + 2)
			Benzenesulfonic acid					
	WCX	Carboxylic acid	Carboxylpropyl					
	SAX	Quaternary ammonium	Trimethylaminopropyl	Electrostatic attraction (anion exchange-ionic acid)	Negative charge analytes	Water or buffer pH < 7 pH = pKa + 2	pH = pKa + 2	Eluent with high ionic strength (pH = pKa-2)
	WAX	Primary, secondary, or tertiary amines						

### Normal phase (NP)

**Fig. 4:**
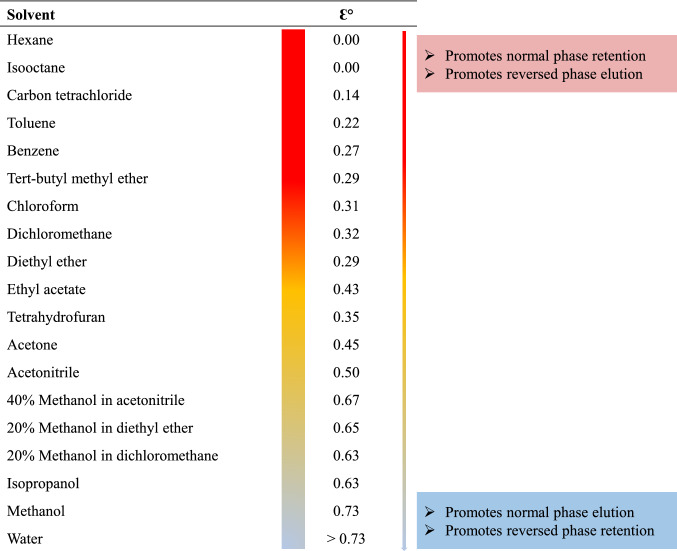
Solvent polarity and eluotropic strength (ɛ°)

NP SPE separates low-molecular polar samples based on the differences in the number and position of functional groups in non-polar matrices, adsorbed by a strong polar stationary phase. The unmodified silica, alumina (Al_2_O_3_), and florisil (Mg_2_SiO_3_) are instances of adsorption normal-phase sorbent. Furthermore, the functionalized silica with polar functional groups (cyano, diol, and amino) exhibit hydrophilic interaction with the solute based on charge-based interactions, hydrogen bonding, π–π, and dipole–dipole interactions. Since NP SPE is commonly used to extract polar analytes from non-aqueous matrices, the tiniest polar components will be eluted first. The extraction using NP relies on the analyte polarity and the functional groups that can interact with the sorbent (hydroxyl, amino, carbonyl, aromatics, double bonds, and groups containing heteroatoms such as O, N, S, and P). To perform the NP SPE, both sample matrices, conditioning solvents, equilibration, and rinse solvent must be non-polar organics to ensure no analyte loss during sample application and sorbent wash. The targeted analytes must elute with high eluotropic strength solvents such as methanol (eluotropic strength, *Ɛ*° = 0.73) or isopropanol (*Ɛ*° = 0.63) (Fig. 4).

### Reversed phase (RP)

RP-SPE used hydrophobic sorbent to quotation non-polar compounds from a polar sample matrix based on adsorption/hydrophobic van der Waals forces [5]. All RP sorbents are silica-based materials with surface modification using hydrophobic groups such as C_2_, C_4_, C_8_, C_18_, C_30_, cyclohexyl, phenyl, and cyano. Due to the polarity range of RP sorbents, the selection of sorbents needs special care. More polar RP sorbents such as phenyl and CN provide better selectivity. Use smaller amounts of elution volume, avoiding the risks of over-drying but increasing the risk of premature analysis rinses during the wash step, requiring a weak washer solvent. However, the more non-polar RP sorbents have a broader analyte retention range, allow for a more potent wash solvent, and may require a plethora in elution volume with a significant risk of insufficient cleanup. All hydrophobic sorbents need activation with a polar organic solvent and then removed with water or buffer. The elution system of a non-polar analyte must be carried out with a non-polar solvent with suitable strength. A mixture of aqueous buffer and polar organic solvent, having appropriate elution strength for polar to semi-polar analytes, leaving the constituents (Fig. 4). Recently, triacontyl bonded silica (C_30_), the latest RP used as packing materials for LC to improve the separation of the geometric isomers and as extraction sorbent for SPE to enrich high hydrophobic analytes from an aqueous sample. C_30_ has better characteristics than C_18_, including long alky chains (hydrophobicity), hydrolytic stability even in highly aqueous conditions, and relatively large particle sizes with sufficient active surface areas. These characteristics exhibit high adsorption capacity [37].

### Ion exchange

Charged polar solutes (acid or base) can be efficiently extracted from polar media, including water and less polar solvents, using a specific mode of extraction known as ion-exchange extraction (IX) [5, 38]. In this case, the isolation mechanism is based on the high-energy electrostatic interaction between the charged functional groups of analytes and sorbent. Thus, the sorbent selection depends on the analyte charge. The cation exchange (CX) column extracts basic analytes (primary, secondary, tertiary, and quaternary amines). In contrast, the anion exchange (AX) column was used to isolate the acidic analytes (carboxylic acid, sulphonic acid, and phosphates). According to the ionic group bonded to the surface, AX and CX can be classified into weak and strong ion exchangers. Strong cation exchangers (SCX) involve a strong acidic functional group, such as an ionized sulfonic acid over the pH range. Weak cation exchange resin (WCX) functionalized with a negatively charged group at high pH and changed to neutral at low pH, like carboxylic acids. Strong anion exchangers (SAX) consist of fully ionized groups, such as quaternary ammonium groups, over the entire pH range. Weak anion exchangers (WAX) have primary, secondary, or tertiary amine moieties ionized at low pH but neutral at high pH (Table 2).

Preparing the sample for AX SPE is necessary because the sample exhibits pH and the lowest ionic strength possible. Therefore, in the case of the CX, the sample pH must be adjusted to two units below the analyte pKa. The pH adjustment should be carried out using strong acid or a powerful buffer to prevent increasing the ionic strength. In contrast, the pH should be increased two units above the pKa using a base or buffer with the AX SPE. In addition, the stationary phase must be conditioned and equilibrated to be in the charged form to interact with the analytes for retention. The SPE cartridge should be conditioned by mixed volumes of water and miscible organic solvent followed by pure water. The cartridge equilibration provides two essential purposes; first, convert the packed materials counter ion to one that is easily exchanged by the analytes. Second, adjust the pH to serve charged groups. A salt solution or buffer can perform the equilibration. The composition of the exchangeable ions depends on their concentration and their affinity with the exchange site. Highly charged ions are preferred over large and weakly charged ions. In CX SPE, the affinity series of the cations are Ba^2+^  > Pb^2+^  > Ag^2+^  > Cu^2+^  > Fe^2+^  > Mg^2+^  > K^+^ = RNH_3_^+^  > NH_4_^+^  > Na^+^  > H^+^. Thus, hydrogen is the lowest affinity cation, and many sorbents can be purchased in the hydrogen form. However, the lowest affinity ions are fluoride and hydroxide in AX affinity series: HSO_4_^−^ > NO_3_^−^ > HSO_3_^−^ > NO_2_^−^ > Br^−^ > Cl^−^ > HCO_3_^−^ > HPO_4_^−^ > HCOO^−^ > CH_3_COO^−^ > F^−^ > OH^−^. On an important note, the sample loading step must be performed at a slow flow rate because the mass transfer kinetics of AX SPE is slower than the RP and NP.

### Mixed mode

Mixed-mode SPE has become very popular in the last decade, exhibiting two or more mode interaction mechanisms such as hydrophobic and ion-exchange functional groups attached to the surface [3, 5]. A hydrophobic group can range from short-chain (i.e., C_2_ group with a high selectivity) to highly retentive (such as a C_18_ group). The IX functionalities can be CX, AX groups, or both in one sorbent. The mixed-mode approach is preferred due to the reproducible ease of binding a single functional group to the silica surface. In addition, different ratios of single active group sorbents can be mixed if other retention properties are necessary. The development of mixed-mode sorbents can provide clean extracts from highly complex interference. The eluent should include non-polar solvents with appropriate buffers, acid, or bases to elute analytes retained in the sorbent by hydrophobic and IX retentive interaction mechanisms.

### Molecularly imprinted polymers (MIPs)

**Fig. 5:**
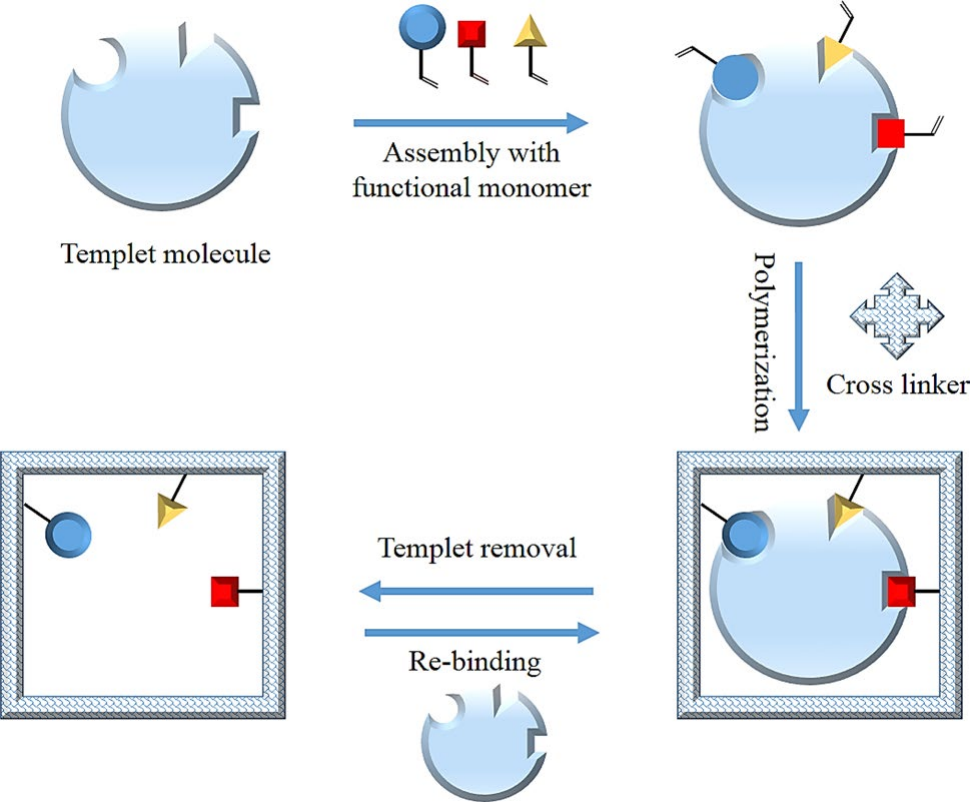
General scheme for MIPs preparation

MIPs are artificial analogs of immunosorbents that were first reported by G Wulf, which are easy to prepare and less broad [39]. The connotation of the imprinting process is the polymerization of the functional monomer and cross-linker (vinyl moieties) in the presence of a templated molecule and pores forming agents. They are typically organic copolymers in a 3D network where a template is trapped with recognition sites. Then the template leaves the cavities sits by washing to complement the size, shape, and molecular interaction. The obtained monolithic polymer is pulverized and then sieved to reap desirable particle sizes to be packed (50–500 mg) into an SPE cartridge (Fig. 5). Moreover, with the development of the preparation process of one put reaction, precipitation, sol–gel multi-step swelling, and polymerization in the presence of surface-active agents were used to obtain spherical particles with the desired size [40, 41]. Nowadays, the assortments of MIP are commercially usable in different areas and applications. Otherwise, the advantages of MIP are the high stability, predetermined recognition ability, higher enrichment rate and selectivity, suitability with a wide range of elution solvents, and low cost [42, 43]. The general steps of MIP SPE include cartridge conditioning by the loading solvent to maximize the MIP interactions with the target molecule in the sample. There are two methods of sample loading involving aqueous loading and organic loading. The aqueous loading utilizes the sample hydrophobicity to incorporate with the polymers. This loading method provides insufficient selectivity and adsorbs the interfering substances. In the organic loading method, low-polarity organic solvents such as acetonitrile, chloroform, dichloromethane, and toluene are used to protect the binding sites and overcome defects. Washing is the most crucial step in MIP SPE. The washing solvents are usually chosen according to the properties of the templates and the interfering contaminates. However, an extremely low-polarity organic solvent is used, such as chloroform, dichloromethane, toluene, or mixtures. To provide a quantitative recovery and high enrichment factor (EF), the target analyte should be eluted using a polar solvent such as trifluoroacetic acid and triethylamine to provide a quantitative recovery and high enrichment factor (EF). The eluant is dried and then re-dissolved in an appropriate solvent for the determination by a specific analytical instrument.

### Restricted access media (RAM)

**Fig. 6:**
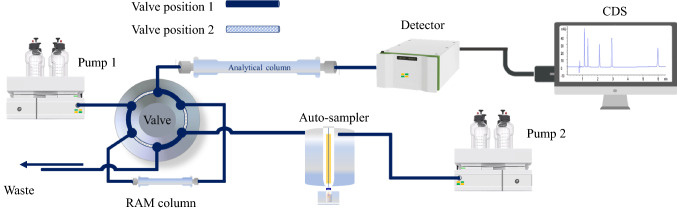
Scheme of the automated on-line SPE-HPLC system

**Fig. 7:**
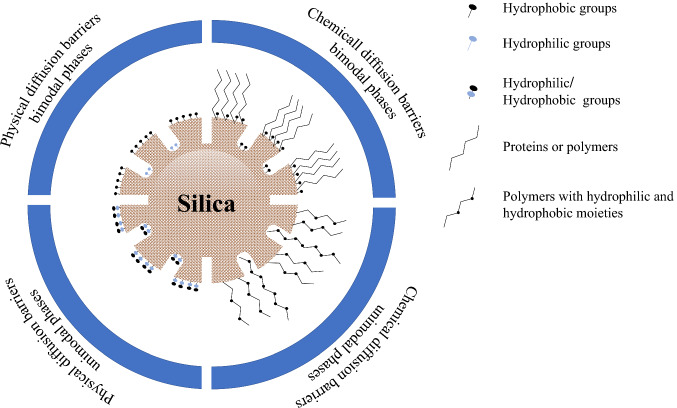
Classification of the restricted silica-based materials

The modern trend in sample preparation includes SPE cartridges and 96-well plates and the on-line injection of the sample using a RAM column (Fig. 6). Desilets et al. first reported this technique to describe new sorbent phases of protein-coated octadecylsilane that enable direct and repetitive injection of unprepared plasma samples into LC [44]. By simultaneous size-exclusion with hydrophobic and IX interactions, RAM phases limit macromolecule access to the stationary phase. By subsequent size-exclusion barriers, the support coated hydrophilic outer coatings eliminate macromolecules from biological matrixes. At the same time, hydrophobic analytes are retained in the interior phase via a partitioning mechanism. Thus, the RAM can be classified based on the nature of exclusion barriers to covalent bond or adsorption or physical and chemical barriers (Fig. 7) [45–47].

The physical barrier, including alkyl-diol-silica sorbent, is the most common RAM material in tread name LiChrospher. Diol groups on the surface of glyceryl propyl particles provide hydrophilic groups with diol moieties that exclude macromolecules through a physical barrier with a pore size of 6 nm. Reverse phase or SCX are used to functionalize the inner surface of the porous silica particles to enrich the target analytes [48]. The diol barriers are commonly used for on-line peptides extraction, and sterols in biological fluids such as serum and plasma are then coupled with two-dimensional RP LC–MS/MS. The SCX-RAM was developed by coating the silica particles using SCX methacrylate monomer (poly(3-sulfopropyl methacrylate) and ethylene di-methacrylate as cross-linker p(SPM/EDMA). Then, a hydrophilic chemical barrier is grafted by glycidyl methacrylate on the p(SPM/EDMA)-grafted silica. Consequentially, the hydrolysis of the epoxy groups to obtain a diol hydrophilic barrier. This kind of development provides the chance to introduce the porous rod structures (monolith), which are more suitable for direct injection of biological fluids [45]. The chemical barriers, including semi-permeable surface (SPS) based on polyoxyethylene polymer bonded to the surface of RP packing materials (phenyl, C_8_, and C_18_). The SPS hydrophilic layer work as a chemical diffusion barrier to restrict protein access to the hydrophobic stationary phase shell [49]. The SPS is commercially available by Regis Technologies.

Protein-coated silica material is composed of porous silica particles shell covered by a protein network of α_1_-acid glycoprotein or bovine serum albumin (BSA) to provide external hydrophilic chemical diffusion barrier macromolecules. In contrast, the hydrophobic groups at the inner surface are responsible for analytes interaction. Silica coated with protein was discovered and used for direct injection of biological fluids. Supports containing hydrophobic groups C_8_ and C_18_ are sold under the trade name BioTrap [50]. Besides the BioTrapMS with a hydrophobic polymer coating coupled to MS.

The mixed functional phase (MFP) consists of both hydrophilic polyoxyethylene and hydrophobic styrene groups embedded in a siloxane polymer coating on a porous silica particle (8 nm) was prepared. As a result, only the hydrophilic, non-adsorption polymer network interacts with matrix components, allowing them to elute within the void volume [51]. Several commercial mixed extraction materials are available with phenyl and C_8_ as hydrophobic moieties or functionalized strong CX groups instead of styrene groups. The MFP is commercialized under Capcell Pak MF’s trade name. Methylcellulose-immobilized on the surface of the silica material to stifle the large molecules (> 3 nm), such as proteins, was developed [47]. However, the surface made as a RAM retains the analytes with various functionalized ligands (C_4_, C_8_, C_18_, SCX, WCX, and SAX).

## Adsorption and extraction parameters

**Fig. 8:**
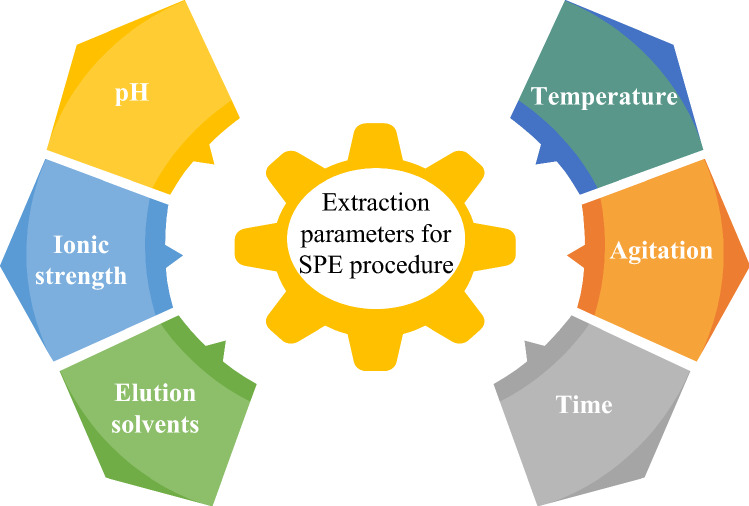
Typical parameters are considered in the development and optimization of SPE methods

**Fig. 9:**
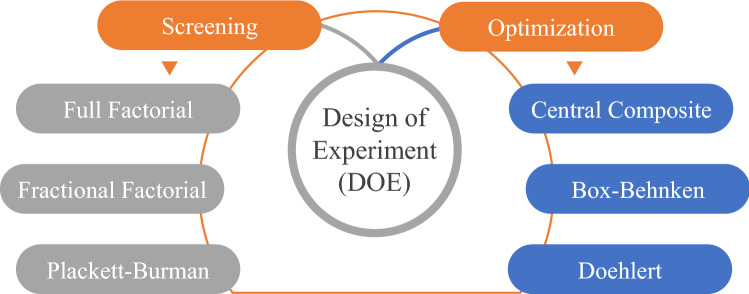
Classification of DOE used in the screening of different factors for optimization of SPE technique

Although the SPE cartridge and other configurations have most development technologies, including different sorbents and retention mechanisms thus, the understanding of SPE requires studying some crucial parameters to select the best conditions for adequate performance and the best possible response of breakthrough. Once the sorbent phase was chosen depending on the solute and matrix properties, it is essential to consider these parameters (Fig. 8) during the optimization procedure [10–12]. There are different strategies to perform the optimum conditions and monitor the influence of the selected factors. The old method is to study one factor at a time and fix the others. Consequently, each factor has an experimental response, which increases the number of experiments with consuming reagents, materials, time, and labor to conduct the optimum condition of the study. Recently, statistical analyses and multivariate tools were developed to perform better during the experiments [11, 52]. A multivariate design of experiments (DOE) became an excellent mechanism to evaluate several factors simultaneously at different levels and to provide a large amount of information in a minimum of experiments, resulting in reduced reagents, materials, time, and effort (Fig. 9).

### pH

**Fig. 10:**
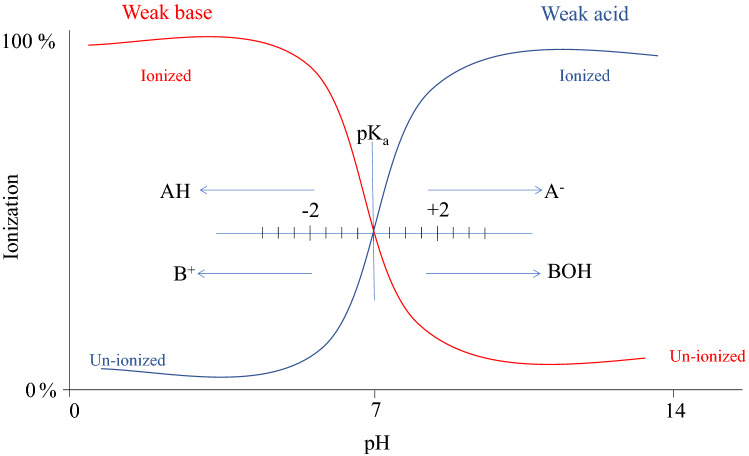
Effect of the pH in the ionization percentage of the hypothetical acid and base

The pH can be one of the most critical factors in controlling the retention and elution of the analyte. Best recoveries are obtained when the sample pH condition provides the analyte in the optimum state for interacting with the sorbent material [53]. The pH can be a source of many problems with different SPE retention mechanisms. In samples loaded in NP SPE, the pH is not usually an issue in the interactions because the elution solvents must be typically non-polar. In the case of the RP mechanism, there is no needs to adjust the sample pH if the analytes are neutral compounds, and retention strengthens under RP conditions. However, the pH of charged ionizable compounds should be adjusted to 2 units above or below the analyte pKa according to the charged group (basic or acidic compounds). So the solute becomes in a neutral state (uncharged compounds), as shown in Fig. 10. Neutralizing base exists at least two pH units above the analyte pKa.

In contrast, the acid analyte becomes neutral at pH below two units than the analyte pKa. An important distinction to keep in mind when employing IX mechanisms is the nature of the charge state of the analyte. Suppose the analyte is charged in all pH ranges, the recommended pairing with a weak ion-exchange. Moreover, if the analyte is charged under certain pH conditions, it is considered a weakly charged compound that must interact with a strong IX sorbent.

A series of polymeric SPE sorbents of styrene-methacrylate and styrene-*N*-vinylpyrrolidone copolymers were evaluated against a neutral pH water sample to recover pharmaceuticals and estrogens at high recovery rates (70–100%) [53]. Pichon et al. found recoveries > 80% for acidic and neutral pesticides extracted jointly from water at pH 7 with the hyper-crosslinked polystyrene-divinylbenzene (PS-DVB) sorbents [54]. They showed that the co-extraction of humic and fulvic acids was significantly reduced at pH 7 compared to extraction at pH 3. In addition, other investigations yielded a recovery of 40% or more of the acidic pharmaceuticals from alkaline seawater (pH 8.3) using the same sorbent [55].

### Ionic strength

The solubility of the analytes in the aqueous samples decreases when the ionic strength is increased by adding salts such as NaCl or Na_2_SO_4_. In this process, salt ions are attracted to water, which will make analytes less available for solvation. Therefore, greater recovery of the analytes may result. Salt concentration effects must always be demonstrated experimentally [56].

### Solvents

Solvent compositions for analytes recovery need to differ from those used in washing solutions. Based on the retention mechanism, an appropriate elution and washing solvent must be chosen (Fig. 4 and Table 2). For solvent selection, *ε*° and the polarity index are very helpful in designing extraction procedures [57]. The polarity index is used to calculate the polarity of a solvent for low-level electrolysis (a measure of the ability of the solvent to act as a proton donor, proton acceptor, or dipole). Typically, an eluotropic series consists of solvents arranged in decreasing order of elution strength for given solutes from a particular adsorbent. A plot of the solvent strength as a function of binary composition is useful for determining the best *ε*° selection. A plot like this does not show linear variations in solvent strength, but it provides an approximation that can be used to develop new methods of SPE. Binary mixtures with *ε*° greater than 0.5 should be considered to dissolve non-polar solutes before adsorption on non-polar adsorbents (e.g., octyl and octadecyl bonded silicas). The choice of an eluting solvent is determined by the relationship of *ε*° and the polarity of the analyte. Almost all polar analytes will be removed from polar adsorbents using methanol as an eluate because of its high *ε*° (0.73). A unique property of methane is that it reacts with both polar and non-polar groups. Methylene chloride (*ε*° = 0.32) often effectively removes non-polar analytes from the non-polar bonding phases. Solvent evaluation is more of an art than a science, despite adhering to the principle of “like dissolves like” [58].

### Temperature

The temperature is a significant factor in optimizing SPE [59]. Increasing the temperature leads to a higher diffusion rate, decreased equilibration time, and increased analyte concentration in the headspace in the case of SPME. In spite of this, extraction recovery decreases as the distribution of analytes between the stationary phase and sample becomes less favorable. Consequently, low temperatures will lead to a long equilibration period and a slower diffusion rate.

### Agitation

The agitation is used to accelerate the adsorption process, especially in SPME and d-SPE [60]. The agitation reduces the equilibration time to the extraction time. Many methods are used to agitate the sample, including magnetic stirring, ultrasonic waves, and vortex. Ultrasound waves, on the other hand, have the effect of increasing temperature in the extraction process, thereby causing the analyte to diffuse between the sorbent material and the sample matrix [61].

### Time

Equilibration time is the main factor affecting extraction efficiency. Thus, the flow rate and dimension of the column are related to the equilibration time [62]. In general, flushing the column with sufficient volumes to obtain enough equilibration. Therefore, the flow rate has to be kept in mind. It has been pointed out that the optimal equilibrium time provides the highest detection limits. Moreover, the increase in extraction yield is greatest in the early stages of extraction and decreases with time. Extraction conducted in a short period of time results in poor repeatability. However, if the extraction time is close to equilibrium, the repeatability will be improved.

### Performance accuracy

The analytical method must be validated using sensitivity, selectivity, accuracy, precision, repeatability, reproducibility, recovery, and enrichment. The precision of an analytical procedure is the closeness of obtained results around individual analyte measurements. The precision can be considered as the SD of multiple aliquots of the same sample. Accuracy refers to the similarity of the obtained result to the actual value. The repeatability means the measurements at the same day precision, intra-day. However, the reproducibility is between laboratories’ precision [12, 63].

The most critical measurement donated to the SPE efficiency in recovering the analytes from the sample. The recovery is defined as the relative amount of analyte measured in the extraction eluent compared to the original sample (Eq. 1):

1 $${\text{Recovery }}\left( {\text{\% }} \right) = \frac{{C_{e} { } \times V_{e} }}{{C_{i} { } \times V_{{\text{i}}} }} \times 100$$

where *C*_*e*_ is the analyte concentration in the total sample extract volume, *V*_*e*_ (Eq. 2), and *C*_*i*_ is the initial sample concentration in the total volume of the original sample V_i_:

2 $$C_{e} = \frac{{C_{s} \times A_{e} }}{{A_{s} }}$$

where *C*_*s*_ is the analyte concentration in the fortified sample. *A*_*e*_ and *A*_*s*_ are the peak area of the sample extract and fortified sample, respectively. Thus, to calculate the recovery, two types of samples will be prepared (fortified with a certain amount of internal standard) and a reference sample in which the analyte is absent. The high recovery percentage indicated that almost all the analysts were recovered, but low recovery does not mean that the chosen sample preparation method is unsuitable for this analyte. Therefore, the EF is the second significant factor that must be measured to identify the SPE performance (Eqs. 3 and 4):

3 $${\text{EF}} = \frac{{C_{e} }}{{C_{i } }}$$

4 $${\text{EF}} = \frac{{V_{i} { } \times {\text{ Recovery}}}}{{V_{e } { } \times { }100}}$$

The EF is the ratio between the extracted concentration and the initial sample before preconcentration. Depending on the recovery, the extracted analyte concentration is always the same or lower than the initial concentration. EF and recovery are strongly correlated to each other. The diluted samples provide an EF of less than 1. However, EF above 1 refers to an excellent enriched concentration when the sample contains a sufficient concentration. EF is affected by sample size, temperature, and solvent evaporation. High EF values are not necessarily associated with sample preparation efficiencies. EF may be achieved by consuming many samples and increasing linearly with the extraction time [64].

## Integration of SPE with analytical chromatographic techniques

Integration, a new paradigm and common terminology in analytical chemistry, combines different parts to form a whole with new distinctive features and functions instead of the other isolated parts. This integration does not necessarily mean the miniaturization of the system but rather how an analytical procedure is done [65]. On the other hand, hyphenation is an on-line blend between a chromatographic separation technique and one or more spectroscopic or spectrometric detection techniques leading to systems such as LC–MS, LC–MS/MS CE-MS, GC–MS, and LC-NMR [66, 67].

Most of these hyphenated techniques would require an initial sample preparation step before separation for analyte enrichment, as in the case of trace analysis. Hence, SPE and SPME can be on-line interfaced with these hyphenated techniques creating much more powerful integrated systems that can be fully automated. The latter provides simplicity and miniaturization of the whole design and is routinely used. This section will focus on some basic operational principles of various hyphenated analytical techniques that have been on-line integrated and automated with SPE or SPME by looking at several literature examples.

### SPE and GC

Techniques involving the on-line coupling of SPE to GC are less common and more complex due to incompatibility between solvents used for SPE with the stationary phases in GC. The need for derivatization and small injection volumes required for GC further hinder this integration. Most automated on-line systems use a six-port valve combined with a drying gas or solvent-vapor exit, which is especially critical in large-volume (LV) transfer GC [68]. The advantages of on-line coupled and automated SPE-GC are considerably reduced analysis time, improved accuracy and precision, high sample throughput, and a less tedious technique. Thoma and co-workers demonstrated these advantages mentioned above by being able to save up to 55–80% of sample preparation and operational costs with the additional improvement in method accuracy, precision, and sensitivity through automation and on-line injection capability of an SPE-LV–GC–MS technique targeting semi-volatile organics in water [69].

An SPE Twin PAL system holding a 96-well SPE plate was interfaced with GC–MS in the literature mentioned above. The upper PAL allowed sorbent cleaning, conditioning, extraction, washing, drying, and elution, while the lower PAL mixed the eluate and standard solutions before injection into the LVI injector. Method LODs achieved using this system were lower than 0.1 µg/L, RSDs were less than 10%, with 70–130% recoveries for reagent water, well water, and tap water matrices.

Prevalent SPE-GC integration techniques involve either manual or partial automation. Full automation mainly relies on robotic systems. For example, Lerch et al. used a RapidTrace SPE workstation combined with a heating block with ten nitrogen-streamed vial positions for the automated SPE before injection into a hot split/splitless port on a 7890GC/5975 MS system [70]. Comprehensive automation between the two, SPE and GC–MS, relied on an *x*–*y*–*z* robotic system that imitated sample dilution, extraction, evaporation, derivatization, and sample injection. Quantitative determination of opioids, cocaine and metabolites from almost 170 authentic serum samples and more than 50 authentic samples of matrices such as heart blood and urine were achieved using this system. LOD and LOQ were comparable to similar manual techniques [71].

Due to related complexities and lack of robustness when SPE is on-line combined with GC, other techniques such as headspace SPME (HS-SPME), fiber SPME, capillary SPME, and microextraction in a packed syringe are used are better suited for on-line GC methods [68]. These microextraction techniques being solvent-free and of small size, are more appealing for interfacing with GC making SPME-GC a popular approach compared to SPE-GC [72]. The fiber configuration of SPME befits the traditional liquid-injection GC syringe allowing for easy automation.

In general, any autosampler capable of performing syringe injection is capable of modification for automated SPME-GC. The first documented automated SPME-GC was by Pawliszyn and co-workers, who modified a syringe autosampler to be SPME capable [73]. This inspired other more prominent names to produce autosamplers capable of SPME integration and automation. In analyzing volatile terpenoids in wine, Williams and Buica compared off-line SPE-GC–MS and on-line HS-SPME-GC–MS methods. While both methods had good linearity, precision accuracy, and LOQs lower than the odor threshold in wine, HS-SPME-GC–MS was more sensitive and had better accuracy [74]. As well to being less tedious and high throughput, it has other advantages [75].

Asides from the fiber configuration, in-tube SPME devices have also been integrated with GC for automation and on-line analyses. Lan et al. developed a fully automated on-line dynamic in-tube extraction GC–MS method for the continuous and quantitative monitoring of volatile organic compounds in the air with remarkable LOQs [76]. When on-line integrated with GC, IT-SPME is less sought-after, with a preference for off-line preconcentration and desorption before analysis [77, 78]. On-line integration of SPE and configurations of SPME, except for fiber SPME, are better applied in LC systems due to the simplicity of the designs and compatibility of solvents used in both systems, as will be seen in the next section.

### SPE and LC

SPE on-line integration with LC systems is much more common compared to GC. The use of single or two six-port automated switching valves enables the integration and automation of SPE with LC systems. Typical 10–30 mm long precolumns with internal diameters of 1–3 mm are used with the standard 4–4.6 mm internal diameter separation columns. The sample volumes used for SPE-LC are ordinarily 1–10 mL, with 10–1000 mL required for environmental samples. This integration is shown by the simple handling of the solvents at the start of the separation since the same or a weaker solvent is used than in the mobile phase [79].

In analyzing cocaine and metabolites in whole blood, Jagerdeo et al. developed a fast, sensitive, integrated, and completely automated SPE-LC–MS system. Two high-pressure dispensing pumps used an automated cartridge exchanger (ACE) with an SPE cartridge module fixated with Hysphere’s MM anion exchange SPE cartridges (10 mm × 2 mm) to provide the solvents for conditioning, equilibration, sample preparation, and cleanup. An additional two connectable 6-way valves were included in this ACE module. The analytes were eluted directly onto a Gemini C6-phenyl analytical column, followed by separation and detection by MS. On top of this method saving time and having a high throughput, it also had a bias of below 7%, precision below 9%, LOD and LOQ were 3–16 ng/mL and 8–47 ng/mL, respectively [80].

Wang et al. used a Zorbax Eclipse XDB-C_8_ (21 mm × 12.5 mm) guard column as the on-line SPE column to determine six groups of lipophilic marine algal toxins from seawater [81]. Seawater was delivered to the SPE column via a quaternary pump and a six-port valve to switch between loading and elution to a UHPLC-MS/MS system. Marasco Júnior et al. used a similar fashioned system of an Oasis HLB SPE column coupled to an LC–MS/MS system to determine pharmaceuticals in wastewater [66]. Most studies use similar setups resulting in high throughput, time-efficient, and less labor-intensive methods with improved speed and reliability [82, 83].

SPE can also be integrated as an interface between hyphenated systems such as LC-SPE-NMR and supercritical fluid extraction (SFC)-SPE-LC. An example of the prior is analyzing organophosphorus products of nerve agents sarin and soman [84]. A Bond Elute NH_2_ SPE cartridge (2 mm × 10 mm) connected with the LC system via a valve was used. In addition, desorption was done manually using a deuterated solvent before injection to NMR mitigating the high intensity elute resonances observed when just LC-NMR is used [85]. In a similar fashion but with some modifications, Bhatia et al. inserted an SPE cartridge between the LC–MS and NMR. They created a UHPLC-QTOF-MS/MS-SPE-NMR integrated system. This system allows for simultaneous monitoring and redirection of 95% of the eluent to an SPE cartridge before on-line elution to NMR using minimal amounts of a deuterated solvent [86]. Such integrations between LC and NMR result in selective purification and concentration of metabolites from mostly complex biological samples [87, 88]. Additional benefits include saving on cost, being less labor-intensive, and requiring minimal operation time [67].

Fiber SPME is the best fit for automation with GC due to the excellent efficiencies attained through thermal desorption of the SPME fiber in the GC injection port instead of LC. However, the automation of fiber SPME and LC was challenging due to an interface issue in developing in-tube SPME [73]. In their development, Eisert and Pawliszyn substituted the injection loop of a commercial HPLC autosampler with a 60 cm capillary tube (0.25 mm, id) containing the extractive material for IT-SPME-HPLC–UV [33]. Using this technique, polar-thermal compounds can be routinely analyzed.

Ishizaki and Kataoka also used a similar approach using a GC capillary column (60 cm × 0.32 mm i.d.) placed between the injection loop and the injection needle of the autosampler for their automated and on-line IT-SPME-LC–MS/MS determination of four tobacco-specific nitrosamines in combusted and heated tobacco products [89]. Herraez-Hernandez’s research group used a TBR-5 column (95% polydimethylsiloxane and 5% polydiphenylsiloxane) 15 cm long, 0.32 mm i.d. and 3 µm coating thickness as the SPME device integrated to nano-LC-DAD, demonstrating its reliability in quantifying contact traces of cannabis [90].

Aside from the open tubular IT-SPME, other configurations involving particles or fibers longitudinally packed inside fused-silica capillaries, polyether ether ketone, polytetrafluoroethylene (PTFE), stainless steel, or copper tubes can also be used for integration with LC systems [34]. Souza et al. reviewed the fiber-IT-SPME-HPLC systems demonstrating their remarkable extraction efficiencies compared to classical IT-SPME but without significant changes in organic solvent consumption and recovery rates [91]. The higher efficiencies result from sample percolation in the narrow coaxial channels leading to a high surface area to sorbent ratio and reduced pressure drops during extraction and desorption. While carryover from sharing common modules can limit the performance of some of these on-line integrated techniques, the benefits that have been outlined outweigh such limitations creating robust SPE/SPME-GC/LC integrated systems.

## SPE applications and future perspectives

### Pharmaceuticals and drugs

SPE technology is widely used with HPLC and GC to analyze and control drug and drug quality during manufacturing. Drug quality control is one of the primary goals of drug manufacturers. Pharmaceutical material and product quality control involve developing specific tests and procedures for raw materials, intermediates, and final products. In addition, performing tests on a topic, compiling results, and regularly submitting them to regulatory authorities. Its identity, efficacy, purity, and quality are continuously evaluated and monitored [92]. However, pharmaceuticals have been recognized as important emerging environmental pollutants, occurring in various environmental sections, including wastewater, groundwater, soil, and even drinking water, reported at trace levels (ng to few µg/L) [93]. Either pharmaceutical can be released into the environment as parent compounds or as metabolites; therefore, monitoring studies should include the parent compounds and their metabolites. Advances in analytical technology have been a significant factor in discovering pharmaceuticals, their metabolites, and their transformation products in environmental matrices. Recent selected SPE applications for extracting and analyzing pharmaceuticals and drugs in different environmental and biological samples are shown in Table 3.

**Table 3:** Recent selected applications of SPE for analysis of pharmaceuticals and drugs in different environmental and biological samples

Adsorbent type	Sample type	Drugs	Extraction technique	Chromatographic system	LOD	References
TiO_2_ nanoparticles and C-nanofibers modified magnetic Fe_3_O_4_ nanospheres (TiO_2_@Fe_3_O_4_@C-NFs)	Drug formulations and surface water samples	Ibuprofen, non-steroidal anti-inflammatory drugs, and azo dye	MSPE	HPLC–DAD	0.95 µg/L	[111]
Graphene oxide-Fe_3_O_4_ nanocomposite	Urine	Morphine, 6-monoacetylmorphine, amphetamine, methamphetamine, codeine, cocaine, dolantin, and benzoylecgonine	MSPE	UPLC-MS/MS	0.02–0.2 µg/L	[112]
Nano-Fe_3_O_4_@Fe-(benzene-1,3,5-tricarboxylic acid)	Water	Bezafibrate, clofibric acid, clofibrate, gemfibrozil, and fenofibrate	DSPE	HPLC–UV–Vis and UPLC-MS/MS	4–99 μg/L	[113]
Molecularly imprinted polymer	Human plasma	Gliclazide	MISPE	HPLC–UV	1 μg/L	[114]
Starch-Mg/Al layered double hydroxide composites	Hospital waste water, river water, sewage treatment plant water, and tablet formulations	Aspirin, mefenamic acid, ketoprofen, N-benzylphenethylamine, diclofenac, and ibuprofen	SPE	GC–MS	4–20 pg/mL	[115]
Fe_3_O_4_/graphene oxide-COOH nanocomposite	Plasma, hospital waste, and river water	Ciprofloxacin, tramadol, and famotidine	MSPE	UV Spectrophotometer	0.005–0.025 mg/L	[116]
Mixed-mode cation exchange sorbent	Eggs	78 Veterinary drugs from different classes	DSPE	UPLC-MS/MS	0.03–0.33 µg/kg	[117]
Magnetic covalent organic framework	Milk	Diclofenac sodium	MSPE	HPLC–UV/MS	10 ng/kg	[118]
Zirconium-based metal–organic framework UiO-66-NH_2_ modified cotton fiber (CF@UiO-66-NH_2_)	Shrimp and fish muscle tissues	Ketoprofen, naproxen, flurbiprofen, diclofenac sodium, and ibuprofen	SPE	UPLC-DAD	0.12–3.5 ng/L	[119]
Cu-based metal–organic framework (MOF-199)	Human plasma and water samples	Non-steroidal anti-inflammatory drugs	SPE	HPLC–UV	0.01–0.02 ng/L	[120]
Strong cation exchange group-bonded silica (SCX, InertSep SCX); a pentafluorophenyl group-bonded silica (PFP, SiliaPrep), and a hydrophilic-lipophilic balanced polymeric sorbent (Oasis PRiME HLB 6 cc Vac)	Ranitidine, metformin, nizatidine, valsartan, and telmisartan drug formulations	*N*-Nitrosodimethylamine	SPE	HPLC–MS/MS	0.2–3 ng/L	[121]
Polystyrene coated Fe_3_O_4_-nanoparticles	Aqueous solution	Antidepressant drugs (amitriptyline HCl, Escitalopram oxalate, and sertraline HCl)	D-µ-SPE	GC–MS	0.49–0.59 ng/L	[122]
Molecularly imprinted polymer	Water	Nevirapine, venlafaxine, methocarbamol, carbamazepine, and etilefrine	SPE	HPLC–MS	0.03–0.31 ng/L	[123]
(Z)-Octadec-9-en-1-aminium tetrachloroferrate (III) ionic liquid	Wastewater and human urine	Clomipramine, ketoprofen, and loperamide	DSPE	GC–MS and HPLC–DAD	2.3–2.9 μg/L	[124]

*SPE* solid-phase extraction, *MSPE* magnetic solid-phase extraction, *DSPE* dispersive solid-phase extraction, *D-µ-SPE* dispersive micro-solid-phase extraction, *MISPE* molecularly imprinted solid-phase extraction, *HPLC* high-pressure liquid chromatography, *UPLC* ultra-pressure liquid chromatography, *GC* gas chromatography, *MS* mass spectrometry, *UV* ultraviolet detector, *DAD* diode array detector, *LOD* limit of detection

At present, HPLC–MS/MS is the analytical technique of choice for the determination of pharmaceuticals in environmental samples due to its high selectivity and sensitivity, allowing the detection of compounds at only a few ng/L or less. The SPE method using a silica cartridge allows the more selective retention of the corticosteroids, which are eluted by varying the ratio of dichloromethane and ethyl acetate. An SPE cartridge method has been described in which corticosteroids were eluted from a Chem Elut^TM26^ cartridge by ethyl acetate:dichloromethane (1:1), onto a silica cartridge before analysis by HPLC [94]. Using off-line SPE and UPLC, an analytical method for the simultaneous determination of seven pharmaceuticals and two metabolites belonging to the non-steroidal anti-inflammatory drugs and analgesic therapeutic groups was developed [1, 62, 95]. Extraction conditions were optimized, considering sorbent material, sample volume, and sample pH parameters. In seawater samples collected along Portugal’s northern coast, this method was successfully applied to determine various pharmaceuticals with a range of LOD values from 0.02 to 8.18 ng/L. Several hundred ng/L of many known pharmaceuticals have been detected, including acetaminophen, ibuprofen, ketoprofen, and hydroxyibuprofen.

A multi-residue method was developed to determine 28 basic/neutral pharmaceuticals and illicit drugs in surface water with quantification by UPLC-MS/MS [96]. SPE used Oasis MCX strong cation exchange mixed-mode polymeric sorbent was chosen for extraction. The influence of matrix-assisted ion suppression and low SPE recovery was tested. Basheer et al. described a porous membrane-protected micro-SPE procedure to extract acidic drugs from wastewater and determine HPLC–UV [97]. The μ-SPE consists of a C_18_ sorbent material held inside a polypropylene membrane. Ketoprofen and ibuprofen were selected as model compounds, and the extraction parameters were optimized. The relative extraction rate ranged between 94 and 112%. The LODs for these target analytes in wastewater ranged from 0.03 to 0.08 μg/L. SPE-HPLC analysis was also applied to analyze various veterinary pharmaceuticals [98]. The recoveries of sulfonamides, fluoroquinolone, and β-lactam ranged from 68.3 to 97.9%, with RSD below 8.4%. In addition, the SPE method allowed simultaneous extraction of the 16 pharmaceuticals using the Waters Oasis HLB at pH 7 with recoveries higher than 75% [99]. The analytes were identified and determined by LC–MS/MS using multiple reaction monitoring (MRM). A multi-residue method based on a bag-SPE technique was evaluated to determine ten pharmaceuticals in surface water near a sewage treatment plant (STP) and along a coastal gradient from an STP effluent [100]. All analyses were performed using UHPLC combined with quadrupole time-of-flight (QTOF) MS. The recoveries were 11–65%, with an RSD of < 16% and inter-day variations of less than 18%. In addition, Patrolecco et al. applied an SPE technique using polymeric Strata X extraction cartridges and HPLC for determining nine drugs in wastewaters and surface waters [101]. The method demonstrated satisfactory accuracy and sensitivity on spiked actual water samples with average recoveries of 65–104% and RSD of 16%. All spiked matrices had LOQs between 10 and 1100 ng/L.

A fast and sensitive multi-analyte/multiclass SPE-LC–MS/MS method was developed and validated to simultaneously analyze 89 pharmaceuticals in influent and effluent wastewater samples [102]. Optimal conditions for SPE in terms of analyte recovery were determined by studying the influence of the mobile phase composition on the sensitivity of the method. All the compounds recovered, on average, between 50 and 120%. Precision, expressed as RSD, was consistently below 15%, and the LOD ranged from 1.06 to 211 ng/L. Evans et al. presented a multi-residue method for analyzing chiral pharmaceuticals, including beta-blockers, antidepressants, amphetamines in wastewater, and digested sludge at the enantiomeric level [103]. The method developed comprises filtration, microwave-assisted extraction, and SPE, followed by chiral LC–MS.

Gika et al. used SPE C_18_ cartridges for extraction and cleanup of thyroid gland hormones and some of their primary metabolites from commercial pharmaceuticals and biological samples (serum, urine, and tissue). The recoveries ranged from 87.1 to 107.6% for serum samples and 92.1–98.7% for urine samples [104]. A selective MIP for ketoprofen was synthesized and applied as an SPE sorbent [105]. The analytical method gave LOD of 0.23, 0.17, and 0.09 μg/L for ketoprofen in wastewater influent, effluent, and deionized water. Water influents and effluents spiked with 5 µg/L of ketoprofen showed 68% recovery, while deionized water showed 114% recovery. Schellen et al. developed a methodology comprised of SPE-LC–MS/MS to determine a wide range of drugs in serum or plasma [106]. The quantification of ten out of eleven medicines in serum or plasma may be easily accomplished. The quantitative tests yielded a 95% recovery rate, a decreased LOQ of 0.2–2.0 ng/mL, excellent precision and accuracy, and good linearity over 2–4 orders of magnitude.

Pre-concentration and cleanup by SPE using Oasis HLB extraction cartridges were designed for simultaneous determination of five anti-inflammatory drugs (acetaminophen, diclofenac, ibuprofen, ketoprofen, and naproxen), an antiepileptic drug (carbamazepine), and a nervous stimulant (caffeine) in wastewater [107]. A diode array detector (DAD) was used for the final analysis of the selected pharmaceutical compounds. With an RSD lower than 15.1%, recovery ranged from 71 to 103%. Furthermore, an anion exchanger-based SPE with 21 phenols and acids derived from sewage influent and effluent was designed and optimized [108]. The phenols and acids were then selectively eluted in separate fractions and were derivatized for GC/MS determination. Recently, TiO_2_ nanoparticles and C-nanofibers modified magnetic Fe_3_O_4_ nanospheres (TiO_2_@Fe_3_O_4_@C-NFs) were prepared and applied in SPE cartridges for accurate sensitive analysis of drugs in biological, pharmaceutical and environmental samples [109, 110]. The main analytical parameters affecting SPE performance packed with the nanomaterials such as pH, sorbent amount, eluent type and volume and sample volume were optimized. The new materials are simple in preparation, high efficiency, and sustainable.

### Biological samples and natural compounds

SPE technology has gained a lot of attention in the broad field of biological sample analysis in fields as diverse as clinical chemistry, forensic science, biomedical and pharmaceutical research [125]. This technique has been employed to extract different kinds of origin of biological samples belonging to humans and animals: whole blood, plasma, serum, urine, feces, tissue, seminal fluid, saliva, and bile. The most crucial advantage of SPE in biological sample analysis is a higher recovery (80–100%) with high reproducibility compared to LLE [111]. The higher analysis recovery makes the assay procedure more sensitive, enabling a smaller sample size to be measured successfully. In addition, biological samples may undergo the SPE procedure directly without any pretreatment, thus simplifying the extraction procedure. Utilizing such a rapid procedure makes it possible to process samples containing volatile analytes such as chlormethiazole [126] and 3-methylindole [127].

Determination of five selected β-receptor antagonists (nadolol, acebutolol, esmolol, oxprenolol, and propranolol) in blood and urine by HPLC after sample preparation via retention on a silica gel sorbent in an SPE cartridge was investigated [128]. Recovery rates were best when the pH range of the samples was 3–7.5. Sample loading, washing and elution conditions, the concentration of antagonists to be extracted, and the type of sorbent used were critical in achieving optimum analytes recovery. By UV-initiated polymerization, non-covalent MIPs of cholesterol were prepared and used as SPE sorbents to directly extract cholesterol from various biological samples (human serum, cow milk, yolk, shrimp, pork, and beef) [129]. The MISPE cartridges were best prepared by conditioning with *n*-hexane, loading with *n*-hexane, washing by *n*-hexane: toluene (9:1), and eluting with chloroform:ethanol: acetic acid (3:1:1). Recoveries ranged from 80.6 to 92.7%, with RSD lower than 9.8%. Bruins et al. optimized the potential of the direct coupling of SPE with MS to analyze clenbuterol in urine samples with an LOD of 100 ng/mL [130]. With mixed-mode cartridges, considerable ion suppression has been obtained. Using cartridges containing 8-µ C_18_-bonded silica, the entire procedure of injecting 1 mL of urine, washing, and desorption was developed. The new UPLC-MS/MS approach was developed to analyze food and biological samples for five different glycopeptide antibiotics after extraction and cleanup by cation exchange SPE [131]. Good linear correlations were obtained for the five-glycopeptide antibiotics in the concentration range of 1.0–20.0 μg/L, a recovery of 83.0–102%, and a LOD of 2.0 μg/L in biological samples with low matrix effects. Magnes et al. performed an SPE on-line coupled to LC–MS/MS to quantify eight polyamines (1,3-diaminopropane, putrescine, cadaverine, *N*-acetyl-putrescine, spermidine, spermine, *N*^1^-acetyl-spermine, and l-ornithine) in various biological samples [132]. The analysis was completed within 4 min and validated using serum samples, making the method highly suitable for routine clinical analysis and high-throughput assays. In urine samples preconcentrated with SPE before LC-FL, 4HN, acrolein, and malondialdehyde, the LODs ranged from 6 to 200 nM [133]. Researchers developed a new magnetic carbon nanotube-based SPE method based on ionic liquid (IL) and magnetic carbon nanotubes (MCNTs) for the extraction and determination of flavonoids in spiked urine samples [134]. Good recoveries with low RSD from 3.5 to 4.9% were obtained, and no interaction occurred by endogenous compounds in human urine. As a model drug, vancomycin was separated from plasma and urine samples on a polypyrrole/graphene nanocomposite in DSPE and detected by HPLC–UV. LOD and LOQ were 0.003 and 0.01 μg/mL, respectively [135]. DSPE was used as an adsorbent of carbamazepine-imprinted surface polymers for separating and preconcentrating carbamazepine in biological samples, using grafted and synthesized anodes of SiO2/graphene oxide [136]. The LOD and LOQ under the optimized conditions were 0.1 and 0.3 μg/L, respectively. Furthermore, the relative recoveries for spiked biological samples were above 85%. μ-SPE was used to extract and clean PCBs from human serum samples [137]. After extraction, samples were analyzed by GC–MS. Good linearity (0.1–100 ng/mL) with determinations ranging from 0.9868 to 0.9992 was obtained. LOD values were varied between 0.003 and 0.047 ng/mL. A combination of SPE and GC was developed to determine phospholipid fatty acids in biological samples [138]. In routine analysis, it proved to be robust and reliable when applied to human plasma and human erythrocytes. Analytical procedure withy high recovery (70–120%) of perfluorinated compounds (perfluoroalkyl sulfonates, perfluoroalkyl sulfonates, perfluorooctanesulfonamide, *N*-ethyl perfluorooctanesulfonamide, *N*-ethyl perfluorooctanesulfonamidoacetate, perfluorocarboxylates, and fluorotelomer carboxylate) in whole blood using OASIS WAX^®^ SPE cartridge was developed [139].

In recent years, MSPE has become increasingly popular for extracting drugs and heavy metals from biological matrices due to several advantages over conventional methods [140]. Silica-coated magnetic nanoparticles modified with γ-mercaptopropyltrimethoxy silane for MSPE of trace amounts of Cd, Cu, Hg, and Pb from biological and environmental samples were designed [141]. By applying an external magnetic field to the aqueous solution, the targeted metal nanoparticles could be separated from it, whereas centrifugation or filtration was not required. The LOD values were 24, 92, 107, and 56 pg/L for Cd, Cu, Hg, and Pb, respectively. The Fe3O4@ZrO2 nanoparticles were prepared by the sol–gel method and used to analyze Cr(III) in seven types of biological and environmental samples by MSPE and FAAS [142]. The adsorption capacity for Cr(III) was 24.5 mg/g with an EF of 25 and LOD of 0.69 ng/mL. Li et al. prepared a new type of 3D, echinus-like magnetic Fe_3_O_4_ @ cobalt(II)-based metal–organic nanotube (Fe_3_O_4_@Co-MONT) yolk-shell microspheres as an absorbent for MSPE of PCBs from environmental water and biological samples [143]. The developed method showed good linearity over 5–1000 ng/L, a LOD of 0.31–0.49 ng/L, and good reproducibility (RSD < 10%). A two-step magnetic retrieval process for chitosan-based luteolin, quercetin, and kaempferol was employed to extract these molecules from urine and serum samples for the first time using the MSPE procedure [144]. Proteins or endogenous compounds caused no interferences, and the LODs for quercetin, luteolin, and kaempferol were 1.0, 0.5 and 0.7 ng/mL in urine samples and 10, 2 and 5 ng/mL in serum samples, respectively. Satisfactory recoveries (90.1–106.5%, 91.1–105.5% and 93.5–108.8% for quercetin, luteolin and kaempferol) in biological samples were obtained. The functionalized MNPs were synthesized and were applied to MSPE of sildenafil and its metabolite, desmethyl sildenafil, from human urine and plasma samples followed by HPLC analysis [145]. In both samples of urine and plasma, the LOD ranged between 0.41 and 0.96 ng/mL. The membrane-selective extraction of steroid hormones with dodecyl-grafted magnetic nanoparticles (C_12_-Fe_3_O_4_) was performed using the MSPE technology [146]. In complex biological samples, nanoparticles provided better recovery, meaning the latter will be more effective in these conditions. An ultrasonic assisted dispersive micro-SPE was used by Behbahani et al. to determine the concentrations of cadmium ions in human urine and blood serum by coating magnetic nanoparticles with polythiophene [147]. For this method, the optimum conditions were pH 7.5, sonication time of 3 min, sorbent amount of 35 mg, HCl; 1.1 mol/L; 360 µL; and 110 sonication time of desorption. Cadmium ions were detected at a LOD and RSD of 0.8 ng/L and 6%, respectively. To selectively extract 8-hydroxy-2′-deoxyguanosine (8-OHdG) using MSPE, a new magnetic aptamer sorbent (Fe_3_O_4_-aptamer MNPs) has been synthesized [148]. HPLC–MS was used to analyze the adsorbed 8-OHdG after selective extraction using the aptamer adsorbent. In addition, the synthesized sorbent presented a high level of biocompatibility and stability, along with specific selectivity and high enrichment capacity. Low LOD (0.01 ng/mL), LOQ (0.03 ng/mL), and wide linear range with a satisfactory *R*^2^ (≥ 0.9992) were obtained. The recoveries of 8-OHdG in the urine samples varied from 82 to 116%.

### Pesticides and environmental pollutants

Since hazardous materials are discharged into the environment, pollution issues have existed for many years, and the pollution exists well beyond permitted limits. In general, hazardous materials refer to any substances, whether naturally occurring or man-made, which have a considerable amount of toxicity and pose a health risk to the public. These include pesticides and heavy metals [149, 150]. These pollutants irritate the young generation, causing chronic diseases that slow their development, as well as causing chronic diseases. Environmental matrices can be analyzed using SPE for the extraction and isolation of trace pollutants [151, 152]. As a result, several analytical methodologies using different types of sorbents have been developed for removing traces of organic contaminants from environmental matrices. Different types of sorbents, from older products to those of the latest, such as immunosorbents (IS) and molecularly imprinted polymers (MIP), have been examined for their advantages and disadvantages.

Agriculture and non-agriculture utilize pesticides and other agrochemicals widely. It has been demonstrated that a number of pesticides can be used effectively against insects, fungi, and weeds, such as organochlorine pesticides (OCPs), organophosphorous pesticides (OPPs), carbamate pesticides (CPs), phenyl urea pesticides (PUPs) and pyrethroid pesticides (PPs). Since these chemicals and residues in foods affect the daily lives of people throughout the world, there has been a lot of attention paid to the health risks associated with their use [153]. Agro-industrial and agricultural samples should be analyzed for residues of these chemicals to find out if they are within defined limits. As a result, a simple, fast, and applicable analytical method is urgently needed. By interacting with the sorbents and analytes individually, pesticides can be extracted, preconcentrated, and purified in the best way. The use of SPE has resulted in the extraction, preconcentration, or purification of a wide variety of pesticides since 1970. An overview of recent SPE applications for extracting and analyzing pesticides in different environmental and biological samples is shown in Table 4. ODS or C_18_, aminopropyl (-NH_2_), and PSA (primary and secondary amine) are the most common sorbents used in SPE cartridges [154, 155]. Since the development of SPE techniques, most have relied on nano-sorbents, including modified silica, graphene, magnetic nanoparticles, and polysaccharides [58, 156]. Recently, the cationic co-polysaccharide chitosan (poly-β-(1 → 4)-2-amino-2-deoxy-D-glucose) and its derivatives became one of the most established and suitable sorbents in separation procedures [10, 12]. El-Nouby et al. developed chitosan–siloxane nano-sorbents (Ch–Si NS) for SPE and removal of abamectin, diazinon, fenamiphos, imidacloprid, lambda-cyhalothrin, methomyl, and thiophanate-methyl pesticides from water samples [12]. In addition, SPME extraction has been adequately used to extract pesticides from food matrixes, with numerous advantages, including the absence of toxic solvents, the short time required, compatibility with chromatographic instruments for separation and detection of analytes, and automated process. It is commonly used for chromatographic (GC and LC) and hyphenated chromatographic–mass spectrometry analyses (GC–MS, LC–MS, GC–MS/MS, and LC–MS/MS).

**Table 4:** Recent selected applications of SPE for analysis of pesticide residues in different samples

Adsorbent type	Sample type	Pesticides	Extraction technique	Chromatographic system	LOD	References
Magnetic amino-modified multiwalled carbon nanotubes	Water	Futriafol, metalaxyl, myclobutanil, napropamide, epoxiconazole, fipronil and diniconazole	MSPE	HPLC–MS/MS	0.3–1.5 ng/L	[180]
Poly[oxycarbonyloxy-1,4-phenylene(1-methylethylidene)-1,4-phenylene]	Fruit juice samples	Chlorpyrifos, ametryn, clodinafop–propargyl, diniconazole, tebuconazole, oxadiazon, and penconazole	DSPE	GC-FID	0.34–1.2 ng/L	[181]
Hydrophilic-lipophilic-balanced magnetic particles	Seawater	96 Multiclass pesticides	MSPE	HPLC–MS/MS	0.13–0.42 ng/L	[182]
Magnetic polymer (*N*-vinyl pyrrolidone-divinyl benzene)	Honey	38 Multiclass pesticides and 5 related metabolites	MSPE	HPLC–MS/MS	0.03–0.1 ng/L	[183]
Hydroxylated microporous organic network	Water and fruit juice samples	12 Triazine herbicides	SPE	HPLC–MS/MS	0.03–0.21 ng/L	[184]
4-Formylphenylboronic acid-modified crosslinked chitosan magnetic nanoparticle	Tea	6 Benzoylurea pesticides	MSPE	HPLC–DAD	0.2–0.7 µg/L	[185]
Fe_3_O_4_@COF	Citrus	Thiamethoxam, imidacloprid, carbendazim and thiabendazole	MSPE	HPLC–UV	0.27–1.22 µg/L	[186]
Magnetic iron (III) oxinate nanocomposite	Pineapple, pomegranate, peach, orange, fresh lemon, cucumber, and sour cherry juices	Chlorpyrifos, haloxyfop–*R*–methyl, oxadiazon, diniconazole, clodinafop–propargyl, fenpropathrin, and fenoxaprop–*P*–ethyl	MDSPE	GC-FID	0.23–0.57 µg/L	[187]
Amino- and carboxyl-functionalized magnetic mesoporous silica	Water	Pyrethroids and neonicotinoids	MSPE	HPLC-VWD	0.02–0.33 μg/L	[188]
Amino-functionalized magnetic covalent organic framework composite	Water, soil and tobacco leaves	Sulfonylurea herbicides	MSPE	HPLC-VWD	0.05–0.14 μg/L	[189]
Metal–Organic Framework MIL-101	Water	Polar phenoxycarboxylic acids	SPE	HPLC-VWD	0.052–0.160 ng/mL	[190]
Mesoporous silica sorbent with gold nanoparticles	Water	20 Organochlorine pesticides	SPE	GC-ECD	0.3–20 ng/L	[191]
Fe_3_O_4_/graphene oxide	Fruits	Malathion	MSPE	Colorimetric analysis	14 µg/L	[192]
C18, Florisil, Oasis HLB, XAD-4, and Chromosorb G/AW-DCMS	Water	39 Multiclass pesticides	D-µ-SPE	GC–MS	0.51 ng/L	[193]
Phenyl-functionalized magnetic nanoparticles	Cabbage	6 Organophosphorus pesticides (sulfotep, diazinon, tolclofos-methyl, chlorpyrifos, isofenphos, and ethion)	MSPE	GC-FID	0.08–0.15 μg/kg	[194]
Magnetic cyclodextrin crosslinked with tetrafluoroterephthalonitrile (Fe_3_O_4_@TFN-CDPs)	Medicinal plants	Azole pesticides	MSPE	HPLC–UV	0.011–0.106 µg/Kg	[195]
Magnetic metal–organic frameworks	Water	Carbamate pesticides	MSPE	HPLC-VWD	0.015–1000 μg/L	[196]
Ti_2_C nanosheets	Fruit juice samples	Triazole pesticides	DSPE	HPLC–MS/MS	0.03–0.3 ng/L	[197]
Zirconium-based metal–organic frameworks	Water and fruit juices	8 Chiral pesticides	MSPE	HPLC–MS/MS	0.10–0.35 ng/L	[198]
Hydroxyl-functional magnetic porous organic polymer	Lemon juice and honey samples	Neonicotinoids (thiamethoxam, imidacloprid, acetamiprid and thiacloprid)	MSPE	HPLC-VWD	0.03–3.0 ng/mL	[199]
Metal Organic Frameworks (A100 Al-based MOFs)	Food and water	Carbaryl	DSPME	UPLC-MS/MS	0.01 mg/L	[200]

*SPE* solid-phase extraction, *MSPE* magnetic solid-phase extraction, *DSPE* dispersive solid-phase extraction, *MDSPE* magnetic dispersive solid-phase extraction, *DSPME* dispersive solid-phase microextraction, *D-µ-SPE* dispersive micro-solid-phase extraction, *HPLC* high-pressure liquid chromatography, *UPLC* ultra-pressure liquid chromatography, *GC* gas chromatography, *MS* mass spectrometry, *UV* ultraviolet detector, *VWD* variable wavelength detector, *DAD* diode array detector, *FID* flame ionization detector, *ECD* electron capture detector, *LOD* limit of detection

A method combining GC–MS, GC-FID, and GC-NPD with the HS-SPME technique was developed by Boyd-Boland and Pawliszyn to analyze 22 nitrogen-containing herbicides, and they applied it to wine samples [157]. Jimenez et al. used a DSPME technique to extract 21 pesticides of different chemical families in honey and selectively analyzed them by GC-ECD [158]. In addition, SPME coupled with GC–MS was used to analyze various pesticide residues found in vegetables and fruits [159]. Pawliszyn et al. developed an automated DSPME-GC–MS method to determine pesticide residues in fruit juices [160]. No operator is necessary for extraction and desorption steps in the automated SPME method with fiber vibration. Moreover, the precision of extraction is substantially improved. SPE combined with electro membrane extraction (SPE-EME) were used for ultra-preconcentration of chlorophenoxy herbicides and determined in environmental samples [161]. In this process, graphene oxide was used as a solid phase, and 8% acetic acid in methanol was used to elute the adsorbed herbicides. High EF values were obtained between 1950 and 2000. The LOQs and MDLs were in the range of 1.0–1.5 and 0.3–0.5 ng/mL, respectively.

A graphitized carbon SPE method was developed to analyze five neonicotinoid insecticides (nitenpyram, thiamethoxam, imidacloprid, acetamiprid, and thiacloprid) in fruits and vegetables [162]. The concentrated elute after LC–MS was then analyzed methanol elution. It was found that bell peppers, cucumbers, eggplants, grapes, grapefruit, Japanese radish, peaches, pears, potatoes, rice, and tomatoes had a recovery rate between 70 and 95% when spiked at 0.1 and 1 mg/kg. In addition, SPE cartridges containing acetamiprid, imidacloprid, thiacloprid, and thiamethoxam were used to analyze neonicotinoids in fruit and vegetable samples [163]. From aqueous-acetone-extracted fruits and vegetables, dichloromethane was used as one-step extraction. Analytes of the residue were performed by LC–MS after part of the eluate was evaporated and the residue was dissolved in methanol. For peach, pear, courgette, celery, and apricot grains, average pesticide recovery ranged from 74.5 to 105% at both spike levels of 0.1 and 1.0 mg/kg.

A cleanup step through a C_18_ Sep-Pak cartridge was developed for the routine multi-residue determination of OCP residues in honey [164]. GC-ECD performed the determination with LOD between 0.05 and 0.20 µg/kg. For the determination of seven pesticide residues in food products using magnetic MWCNT SPE, Zhao et al. developed a low-cost and highly efficient process based on ultrasound-assisted deep eutectic solvent extraction [165]. For high extraction efficiencies, diverse solvents were tested. Recovery rates ranged between 76.09 and 97.96%, with a RSD between 0.13 and 10.05%. The SPE-NH_2_ method was used in another study to extract and clean 18 pesticides belonging to various chemical classes from apples, together with GC for determining the amount of pesticide remaining [166]. Using MS detection, recoveries at 5 µg/kg were > 90%, except for dimethoate and captan (77.7% and 46.4%, respectively). To determine OCP residues in seaweed samples, microwave-assisted micellar extraction and SPME/SPE procedures were complemented with microwave-assisted micellar extraction [167]. When using LC-PDA detection, SPME and SPE both achieved excellent results. The average recovery for SPME varied between 80.5 and 104.3%. The average recovery for SPE varied from 73.9 to 111.5%. Results from the Soxhlet extraction procedure were compared with those from SPE. SPE cartridges embedded with HLB copolymers and GC–MS were also used to quantify pesticide concentrations in Northern Lebanon after OCPs were isolated and trace-enrichment was carried out from water samples [168].

The presence of OCPs, OPPs, and pyrethroid residues in green tea is a widespread problem requiring attention worldwide [169]. Using magnesium aluminum double oxide (Mg–Al–LDO) combined with graphitized carbon black (GCB) as a sorbent, 15 pesticide residues were isolated. The combination of Mg–Al–LDO and GCB, sorbent, eluting solvent type, volume, and type of solvent were considered when optimizing the experimental conditions. The recoveries at three spiked concentrations (10, 100, and 500 ng/g) for fenthion, *P*,*P′*-DDE, *O*,*P′*-DDT, *P*,*P′*-DDD, and bifenthrin, ranged from 71.1 to 119.0%. Six water samples from the Lagos lagoon were collected, extracted using SPE, and analyzed by GC [170]. Chrodane was quantified from 0.006 to 0.950 µg/L, heptachlor at 0.067 µg/L, methoxychlor at 0.123 µg/L, hexachlorobenzene from 0.015 to 0.774 µg/L, endosulfan from 0.015 to 0.996 µg/L, and dichlorodiphenyltrichloroethane from 0.012 to 0.910 µg/L. GC–MS analysis of apple juice residues using graphene-based SPE was developed to determine the OPS residues contained therein [171]. After dilution with water, the juice was loaded directly onto the cartridge. Several factors affecting the extraction were investigated, including the type of extraction, washing solution, and sample pH. Under optimized conditions, the analytes were detected with excellent LOQ of 0.15–1.18 ng/mL, and the average recoveries ranged from 69.8 to 106.2%. For the extraction and cleanup of 15 OPs, including their metabolites from water, Gonzalez-Curbelo et al. developed a DSPE method that utilizes MWCNTs as the extraction sorbent [172]. Sample volume, MWCNT amount, and eluent volume were studied as factors that affect enrichment efficiency. In order to validate an optimized method, matrix-matched calibration, recovery, precision, and accuracy measurements were performed for the three analyzed samples. A 67–107% absolute recovery was achieved for all three samples. The measurement of OP residues in food plants and fruit beverages was achieved using a DSPME-GC coupled with FPD or MS [173, 174]. The method was selective and reproducible, and LODs were below 2 μg/kg for all pesticides.

Anastassiades et al. designed an SPE method to extract traces of multi-residue pesticides by dispersive liquid–liquid microextraction (DLLME) and measure their concentration by GC–MS [175]. The proposed method achieved good linearity (*R*^2^ > 0.9915) over 1–10,000 ng/kg by considering both elution and extraction solvents, breakthrough volume, salt addition, and extraction time. The authors’ method produces preconcentration factors between 2362 and 10,593 for 100 mL sample solutions. They also compared the approach to others and found that SPE-DLLME has a higher extraction efficiency and a higher preconcentration factor.

The pesticides ametryn, atrazine, diuron, and fipronil were extracted from the water samples from the Feijão River (Brazil) by SPE and then analyzed by LC-DAD [176]. The recovery was in the range of 90–95%. In the SPE cleanup process, followed by GC–MS, 88 pesticide residues in berry fruits, including raspberry, strawberry, blueberry, and grape, were quantified [177]. As elution solvent, acetonitrile–toluene (3:1) was used with the Envi-Carb cartridge coupled with the NH_2_-LC cartridge. The correlation coefficients of all three pesticides rose above 0.99 at the linear ranges. Recoveries ranged from 63 to 137% between the low, middle, and high three fortification levels.

Graphene aerogel (GA), a typical kind of 3D macroscopic assembly that exhibits interesting characteristics, was discovered as an efficient SPE adsorbent for extracting and removing different pollutants from environmental samples [178]. The adsorption properties of GA for bisphenol A (BPA) showed high efficiency due to its efficient mass transfer, multiple adsorption sites, and good retention of analytes. The obtained cartridge was applied to separate different pollutants from water samples. By optimizing several parameters affecting recovery rates on endocrine-disrupting chemicals and PCBs, LODs were found between 0.01 and 0.11 ng/mL and 0.19–1.53 ng/L for the two series of compounds. Furthermore, all the analytes were recovered in 76.3 to 112.5% based on the satisfied sensitivity. SPE coupled with functionalized magnetic nanoparticles (Fe_3_O_4_@SiO_2_/β-CD) was developed for the extraction of BPA and diethylstilbestrol (DES) from water samples [179]. The sample's volume, pH, adsorption time, and desorption condition were optimized for SPE extraction. The extraction was completed in 25 min under the following conditions: 250 mL water sample, 0.1 g sorbent, and elution with methanol (3 mL with 1% acetic acid). The developed method showed spiked recovery rates ranging from 80 to 100%, and LODs were 20.0 and 23.0 ng/L for BPA and DES, respectively.

A low concentration of metals and a high concentration of matrix components make it challenging to measure rare-earth elements in environmental samples without preconcentration and separation [201]. In addition to the high EF, rapid phase separation, and combination capabilities, an SPE technique with solid sorbents offers many possibilities for detection. As a result of their research, Tuzen and Soylak proposed a system for speciation of Cr(III) and Cr(VI) in samples [202]. The procedure is based on Cr(III) adsorption on the Chromosorb-108 resin. It was carried out by analyzing the various parameters of the sample. In addition, Chromosorb-106 resin was used to extract trace cadmium and lead ions from water samples. The resulting 2-naphthol recovered up to 95% [203]. The influences of various factors, including the pH of the model solutions and the concentration of PAN, were also studied. The SPE was also developed to separate Cd(II) from water samples [204]. On the Duolite XAD-761 resin, Cd(II) is adsorbed as the 4-(2-pyridylazo) resorcinol complex. In addition, the SPE was applied to MWNTs to separate and preconcentrate Cu(II) before its determination using atomic absorption spectroscopy (AAS) [205]. The preconcentration factor was determined to be 60 based on the results. To optimize the procedure, all analytical parameters, such as pH of the solution, eluent type, sample volume, and eluent flow rates, were considered. SPE cartridges were used to extract Cu(II), Cd(II), Pb(II), Zn(II), Ni(II), and Co(II) ions from multiwalled carbon nanotubes (MWCNTs) [206]. The effects of various experimental parameters on the properties of multiple solutions were studied.

A column filled with Amberlite XAD-2000 resin was used to separate metal ions then the analytes retained on the resin were eluted by 1 mol/L nitric acid in acetone and determined by AAS [207]. Analytical parameters such as the pH of a sample solution, ligand amount, solvent volume, and flow rate were investigated. An Amberlite XAD-2010 resin was developed for preconcentration of Mn(II), Co(II), Ni(II), Cu(II), Cd(II), and Pb(II) ions based on their complex formation with sodium diethyldithiocarbamate before AAS determination [208]. In addition, a simple and sensitive SPE procedure on Amberlite XAD-1180 resin was used for the determination of Cr(III), Mn(II), Co(II), and Ni(II), at trace levels by AAS with recoveries greater than 95% and no influences were observed from the matrix ions of table salt samples [209].

An MSPE with bismuthiol-II-immobilized magnetic nanoparticles was developed for the separation/preconcentration of trace amounts of Cr, Cu, and Pb from environmental samples [210]. Optimal conditions were established, and the LOD for Cr, Cu, and Pb with EFs of 96, 95, and 87 were 0.043, 0.058, and 0.085 ng/mL. Yilmaz and Soylak presented the SPE cartridge packed with MWCNTs to extract Cd, Pb, Ni, Cu, and Zn at trace levels [211]. The metals retained on the MWCNTs at pH 6.5 as α-benzoin oxime complexes were eluted by 10 mL 2 M HNO_3_ in acetone. The preconcentration factor was found to be 50, and the LODs for Cd(II), Pb(II), Ni(II), Cu(II), and Zn(II) were 1.7, 5.5, 6.0, 2.3, and 2.4 μg/L, respectively. In water, soil, and sediment samples, a simple and efficient method to identify Cr(VI) and Cr(III), separate and preconcentrate them was optimized [212]. Cr(VI) has been separated from Cr(III) and preconcentrated as Cr(III)-diphenylcarbazone complex using Ambersorb-563 resin and determined by spectrophotometric method at 540 nm. The presented procedure was successfully applied for the Cr(III) in various environmental samples. In addition, 1-(2-pyridylazo) 2-naphtol impregnated Ambersorb 563 resin was used as SPE of Cu, Ni, Cd, Pb, Cr and Co ions in aqueous solutions prior to their AAS determinations [213].

### Food and beverage

SPE has been successfully used to extract many different volatile compounds from various foods for analytical purposes [214]. Table 5 presents a number of recent analytical methods developed using SPE for organic compounds and heavy metals analysis in food and beverages. Food structures represent a complex matrix and can be formed into different physical phases, such as solid, viscous, or liquid. Therefore, the sample preparation step is essential in identifying specific compounds in foods. SPE provides many opportunities to analyze various food samples, improve, and advance. The diversity of sorbent types makes SPE one of the best choices for specific sample preparation needs in food analysis [1].

**Table 5:** Recent selected applications of SPE for analysis of food and beverage

Adsorbent type	Sample type	Compounds	Extraction technique	Chromatographic system	LOD	References
Covalent organic framework (COF-(1,3,5-trimethylphloroglucinol, benzidine)/Fe_3_O_4_)	Beer, juice drink, carbonated drink, milk-containing beverage, alcoholic beverage, tea beverage, fresh-made milk tea and solid beverage	Phthalate esters	MSPE	GC–MS/MS	0.005–2.75 μg/L	[235]
Strong cation exchange SPE Oasis cartridges MCX	Carbonated drinks, beers and energy drinks)	2-Methylimidazole and 4-methylimidazole	SPE	HPLC–UV	3.8–5 ng/L	[236]
Magnetic metal organic framework composite (Fe3O4@MOF-808)	Tea beverages and juice samples	Benzoylurea insecticides	MSPE	HPLC–UV	0.04–0.15 ng/L	[237]
Porous composite of metal–organic and covalent organic frameworks (Fe_3_O_4_@TAPB-COF@ZIF-8)	Functional beverages	Bisphenols	MSPE	HPLC–UV	0.04–0.05 ng/L	[238]
Magnetic ZnFe2O4 nanotubes	Beverage samples	Heavy metals (Mn II and MnVII)	MDMSPE	FAAS and AT-FAAS	0.005–0.007 ng/L	[239]
Molecularly imprinted polymer	Non-dairy beverages	Aflatoxins (B1 and B2)	MIMSPE	HPLC–MS/MS	0.085–0.21 μg/L	[240]
Zeolitic imidazolate framework-8/ fluorinated graphene coated SiO_2_ composites	Environmental and food samples	Chlorophenols	PT-SPE	HPLC–UV	2–20 ng/L	[241]
Graphene oxide composite microspheres	Environmental water samples	Bisphenol endocrine disruptors (bisphenol A, bisphenol B, bisphenol AF, and tetrabromobisphenol A)	DSPE	HPLC–MS/MS	0.02–0.11 μg/L	[242]
Silica-based C_18_ sorbent	Commercial food drinks: fruit juices and soft drinks	Benzoic and sorbic acids	SPE	HPLC–DAD	0.18–0.87 μg/L	[243]
MgO–SnO–Zeolite composites	Wastewater samples	Heavy metals (Cd, Cr, Mn, Ni, and Zn)	DSPE	ICP-OES	0.08–0.45 μg/L	[244]
3D N-doped magnetic porous carbon spheres	Food samples (orange juice and beer)	Biogenic amines (tryptamine, phenethylamine, histamine, tyramine, spermidine, and spermine	MSPE	HPLC–UV	0.06–0.07 ng/L	[245]

*SPE* solid-phase extraction, *MSPE* magnetic solid-phase extraction, *MDMSPE* magnetic dispersive micro-solid-phase extraction, *DSPE* dispersive solid-phase extraction, *MIMSPE* molecularly imprinted micro-solid extraction, *PT-SPE* pipette tip solid-phase extraction, *HPLC* high-pressure liquid chromatography, *GC* gas chromatography, *MS* mass spectrometry, *UV* ultraviolet detector, *DAD* diode array detector, *FAAS* flame atomic absorption spectrometry, *AT-FAAS* atom trapping-flame atomic absorption spectrometry, *ICP-OES* inductively coupled plasma-optical emission spectroscopy, *LOD* limit of detection

This technique is widely used to extract amines, acrylamides, heavy metals, pesticides, mycotoxins, sterols, anthocyanins, folates, and antibiotics in different food samples [214]. Aliphatic, aromatic, heterocyclic, and heterocyclic aromatic amines are the different amine groups found in various food matrixes. Still, the determination of these trace level compounds is problematic because of their high volatility and polarity structures, high solubility in water, and relative basic character [215, 216]. Several food products, especially protein-rich foods (e.g., cooked meat or fish) and fermented drinks (e.g., beer, wine) contain amines, which increase cancer risk [217].

SPE application for acrylamide detection in food samples is a well-documented topic in the literature [218, 219]. SPME has also been paid attention to in detecting acrylamide [220]. For the extraction of acrylamide from coffee beans, an extraction method based on headspace SPME was developed [221]. With commercial SPME fiber-coated polydimethylsiloxane (PDMS), acrylamide was silylated with N,O-bis(trimethylsilyl) trifluoroacetamide, and its concentration was subsequently assessed with GC–MS. This method has a limit of quantification of acrylamide of 3 µg/kg with good reproducibility (RSD: 2.6%), which was under the EU's guidelines for determining the concentration of AA in food [222]. Based on a green synthesis strategy for DMIP synthesis of propanamide as a dummy template molecule, novel DMIPs were prepared under mild conditions. The DMIPs were used to quantify acrylamide in biscuit samples using MSPE and HPLC [223]. MSPE efficiency was examined as a DMIP dose, sample solution pH, extraction time, and desorption solvent. Excellent linearity for acrylamide was obtained in 5.0–5000.0 µg/kg. The LOD and LOQ were 1.3 µg/kg and 4.4 µg/kg, respectively. Based on the results of these studies, it appears that an eco-friendly approach can be used to handle complex matrixes in order to provide highly effective sample pretreatment and targeted analyte determination.

In order to determine sterol levels in food samples, a multi-step process is required, including saponification, purification, and GC or HPLC analysis. However, SPE could be an alternative to cholesterol analysis, especially since it is faster and simpler [224]. Russo et al. developed an SPE-GC method for cholesterol analysis in animal fats. The study results revealed that fat samples containing at least 0.8 µg/mg of cholesterol could be analyzed effectively by this method and that it was a viable alternative to saponification. Furthermore, the SPE technique coupled with supercritical CO_2_ extraction could provide a viable alternative to the traditional Soxhlet method for determining free cholesterol in foods containing eggs [225].

As a prophylactic and therapeutic antibiotic, tetracyclines are widely used in veterinary medicine. They have led to concerns about the contamination of animal products intended for human consumption. For the analysis of seven tetracycline antibiotics in milk, Lock et al. developed a new SPME/LC–MS method in combination with the SPME method [226]. After direct immersion with CW-templated resin fiber, the fiber was transferred to a desorption chamber pre-filled with a mobile phase. A 100 ppb level of tetracycline in milk was successfully analyzed with 4 to 40 ng/mL LOD.

Foods can also contain mutagenic or carcinogenic compounds generated during the preparation and storage. Sen et al. used an SPME technique to analyze *N*-nitrosamines in smoked ham [227]. *N*-Nitrosodibenzyl amine was selectively detected at a concentration of 39.8 μg/kg ham by GC-thermal energy analysis, and the LOD value was in the range of 1–3 μg/kg. Kataoka and Pawliszyn developed in-tube SPME coupled with LC–MS to analyze carcinogenic heterocyclic amines [228]. This method is simple, rapid and automatic and was successfully applied to the analysis of food samples.

The SPE method is of great interest as a purification tool in anthocyanin extraction procedures [229, 230]. Denev et al. obtained anthocyanin-rich extracts with 94.4% of the sugars and 88.5% of the acids separated through SPE methods [229]. Cation exchange and reverse phase SPE were combined by He and Giusti and compared with three previously-used methods such as C_18_, HLB, and LH-20 [231]. The results showed that this method contained high purity of anthocyanins and extensive impurity removal capacity.

The use of the SPE reagent N-benzoyl-N-phenylhydroxylamine and the adsorbent Amberlite XAD-1180 was used to extract Cu(II) and Fe(III) from different food samples [232]. The determination by AAS showed that the cereals contained Cu(II) concentrations ranging from 1.01 to 5.81 µg/g. Vegetables and fruits had concentrations ranging from 0.40 to 9.67 µg/g. In contrast, Fe concentrations ranged from 7.48 to 34.3 µg/g in cereals, 5.74–260 µg/g in vegetables and fruits, and from 1.63 to 5.12 µg/g in beverage samples. A highly sensitive SPE method was developed to determine the amount of iron in water, soil, and botanical materials for the spectrophotometric determination of iron(II) with a recovery range of 98.71–101.51% [233]. Compared with parameters obtained without using the SPE method, the LOD and LOQ were 1.98 and 6.0 ng/mL, respectively. ALOthman et al. impregnated MWCNTs into 4-(2-thiazolylazo)resorcinol and used it for the separation and preconcentration of Cd(II), Pb(II), Zn(II), and Ni(II) from food samples [234]. In this study, acetic acid was used as the eluent, and the analytes were quantitatively recovered at pH 7.0. The effects of pH, flow rate, eluent type, and sample volume were studied. A positive correlation was found between the results of the test and the certified data of the reference materials.

## Conclusion

SPE is a widespread sample preparation technique because it is characterized by ease of operation, low cost, low solvent consumption, speed, and high enrichment. In the future, class-specific sorbents should be developed for the isolation and preconcentration of target analytes in complex matrices. Modern chromatographic instruments such as GC–MS/MS and LC–MS/MS are also expected to benefit when linked with modern technology, thus replacing tedious trial-and-error procedures common today. As a result of these advances, some laboratories will analyze several samples simultaneously or monitor process variables round the clock. Since SPE techniques have a high degree of flexibility, they are commonly used to analyze foods, environmental samples, drugs, and biological samples containing volatile and non-volatile compounds. As SPE is developed in miniaturized forms, it will be integrated with chromatographic and spectroscopic systems on-line. Consequently, a smaller sample will be required, and the sample throughput will be higher. Currently, most research is focused on the development of high-capacity and high-selectivity sorbent materials. Nanoparticles and sol–gel coatings are particularly noteworthy among the sorbents for miniaturized SPE formats. Finally, using SPE techniques in the initial steps of chromatographic analysis reduces the negative impact on the environment and health of laboratory workers, providing an environmentally friendly method of analysis.
